# On quality standards and the timing of pharmaceutical investment

**DOI:** 10.3389/fpubh.2025.1738084

**Published:** 2026-01-21

**Authors:** Wei Wei

**Affiliations:** International College, Guangzhou College of Commerce, Guangzhou, China

**Keywords:** health authority, optimal stopping, pharmaceutical investment, quality standards, real options, social planner

## Abstract

**Introduction:**

This study analyzes how a health authority's quality standard influences a pharmaceutical firm's R&D investment timing, comparing this decentralized process with a social planner's integrated approach.

**Methods:**

Drug quality evolves as a geometric Brownian motion, accelerating after irreversible investment. Using real options and optimal stopping theory, we model a sequential game between the regulator and firm, and a social planner who jointly sets both the investment threshold and quality standard.

**Results:**

The social planner invests earlier and achieves a higher total value than the combined payoff in the decentralized setting. Increasing the firm's remuneration can accelerate investment, but may delay commercialization if quality standards are set too high.

**Discussion:**

Decentralization creates inefficiencies through constrained optimization, whereas the social planner's unconstrained approach maximizes welfare. Remuneration design is a key policy tool for aligning incentives, particularly for neglected diseases that require timely solutions.

## 1 Introduction

This study analyzes a central issue in drug innovation policy: how a health authority's quality standard for a new drug affects a pharmaceutical firm's R&D investment timing under uncertainty. We framed this as a strategic interaction problem, modeled through a sequential game between a regulator (who sets the standard) and a firm (who decides when to invest). To assess the efficiency of this decentralized process, we compare it with the benchmark of a social planner who integrates both the investment timing and standard-setting decisions.

The incentive misalignment of this decentralized structure is particularly relevant for fighting Neglected Tropical Diseases (NTDs). These diseases, defined by the WHO, are causing severe health and economic impacts on more than 1.74 billion people,^1^ primarily in the poorest developing regions. Historically, there has been insufficient private investment in R&D for NTDs due to a perceived lack of a profitable market (1). To address this, public-sector actors and partnerships have employed pull mechanisms such as Advance Purchase Commitments (APCs) (2), (3), (4). These contracts specify not only a price (remuneration) but also a quality or efficacy threshold that the drug must meet. Thus, the question of how a publicly-set quality standard shapes private investment timing is not only theoretical, but is a question in designing effective incentives for diseases of poverty.

While APCs are recognized as a key tool to stimulate R&D for neglected diseases (5), their design involves critical tradeoffs (6). The level of remuneration must balance compensating the firm for risk with fiscal constraints for the funder. Similarly, the mandated quality standard must balance the requirement for a highly effective drug with the need to encourage timely development. Recent analyses of incentive mechanisms, including APCs for vaccines and antibiotics, continue to highlight this challenge of balancing cost, quality, and speed (7, 8). An alternative approach is direct government-led development, though evidence suggests that creating a conducive innovation ecosystem can be more effective than heavy-handed intervention (9). However, this approach may not be sufficient if the immediate priority is to rapidly produce high-quality drugs rather than to foster a self-sustaining market in the long term.

This study contributes by introducing a formal microeconomic model that captures the essential dynamic and strategic elements of this problem: the irreversible nature of R&D investment, the stochastic evolution of drug quality, and the sequential decision-making between the public and private sectors. More specifically, the pharmaceutical firm makes decisions regarding the timing of investing in a sunk cost to initiate a research and development (R&D) project. The quality of the drug resulting from this research evolves stochastically over time. Upon reaching a predetermined quality threshold set by the health authority, the firm gains the opportunity to commercialize the drug and receive an *ex ante* known remuneration for providing it to the health authority. The health authority's payoff from the drug is determined by cumulative population health benefits, while its costs include the remuneration paid to the pharmaceutical firm. In our benchmark scenario, we introduced an integrated social planner who makes joint decisions regarding both the timing of investment and the quality threshold. This setup enables us to compare outcomes under different decision-making frameworks and evaluate the efficiency of coordination between the pharmaceutical firm and the health authority in achieving societal welfare objectives.

This study uses techniques from optimal stopping theory to solve the pharmaceutical firm's timing of investment, aligning it with the literature on real options (10). A considerable amount of literature is dedicated to studying investment under uncertainty across multiple industries. A handful of papers incorporate optimal timing, as evidenced by works such as Sarkar (11), Sarkar and Zhang (12), Thijssen (13), Thijssen (14), Tsekrekos and Yannacopoulos (15), and Huberts and Thijssen (16). In this study, we delve into a mechanism design problem intricately tied to the timing of an agent's decision, in this instance, the investment decision of a pharmaceutical firm. More importantly, we compare the social planner benchmark with the “decentralized” game. It draws parallels to the concept of vertical control discussed in the industrial organization literature, as outlined by Tirole (17). In this context, the health authority assumes the role of the upstream firm, while the pharmaceutical firm represents the downstream firm within the decentralized game.

There are four main findings of this study. First, we demonstrate that the optimal investment threshold under the social planner's benchmark is strictly lower than the investment threshold in the decentralized project. This indicates that a social planner would invariably initiate R&D efforts sooner than the pharmaceutical firm operating independently. Second, in scenarios where the budget for remuneration is constrained, and the sunk costs of the project remain relatively low, the social planner tends to select a higher-quality standard than the health authority. Conversely, when sufficient funding is available to afford higher remuneration, the level of remuneration becomes a viable tool for adjusting the quality standard of the drug. However, raising the remuneration may delay commercialization. Finally, the analysis reveals that the social planner's approach generates more value than the combined efforts of the health authority and the pharmaceutical firm. Importantly, this outcome remains consistent across various model parameters, including the discount rate, growth rate, and uncertainty levels.

The remainder of this study is organized as follows. Section 2 grounds the model in practice and introduces a simple game between a health authority and a pharmaceutical firm. Section 3 discusses the optimal stopping problem of the integrated social planner. In Section 4, we compare the thresholds and values of the two projects, shedding light on the implications of different decision-making frameworks. Section 5 offers concluding remarks summarizing the key findings and policy implications of the study.

## 2 Model framework and real-world contex

### 2.1 Interpreting the model in practice

Before presenting the formal game, we clarify the real-world counterparts of our modeling constructs. The decentralized framework captures the essential strategic interplay common in many health systems: a health authority (HA), acting as both regulator and payer (e.g., akin to the U.S. FDA/CMS or China's NMPA/Healthcare Security Administration), sets a minimum quality standard for market approval or reimbursement. This standard reflects technical requirements for safety and efficacy. A pharmaceutical firm (PF) then decides when to commit its own resources to R&D in response to this regulatory hurdle. This separation of decision-makers is the norm. In contrast (the benchmark case), the social planner (SP) represents an idealized, fully integrated decision-maker, analogous to a mission-driven, publicly funded research program (e.g., the U.S. BARDA or a dedicated government task force for a pandemic) that internalizes both development costs and population health benefits. As for the model environment, the drug quality evolves stochastically, which is a parsimonious representation of the technical uncertainty inherent in R&D. The acceleration in growth rate post-investment (μ_2_>μ_1_) reflects the intensified effort and resource allocation after a firm or planner commits to full-scale development.

Key parameters in our model are motivated by empirical realities and policy variables:

**Quality** (*q*_*t*_)**:** A composite index of drug efficacy and safety, the primary target of regulatory review. **Standards (**$q_{c}^{A}$, $q_{I}^{A}$**):** The minimum q required for the HA to approve/pay, or the SP to deploy the drug. In practice, these are informed by health technology assessment (HTA) and comparator therapies.

**Remuneration (R):** The (often contractually agreed) payment flow from the HA to the PF for a successful drug. This mirrors prices set in Advance Purchase Commitments (APCs) or negotiated in national reimbursement drug lists (e.g., China's NRDL negotiations), where price is explicitly linked to assessed clinical value. **Health benefit (K):** Converts quality into monetary health gains, reflecting the payer's willingness-to-pay for health outcomes. **Costs (I, C):** The sunk R&D cost and marginal production cost, respectively. **Uncertainty (σ):** Volatility in the quality process, capturing the unpredictable nature of scientific discovery and clinical trial results.

This setup allows us to analyze how the regulatory/payment threshold ($q_{c}^{A}$) and the financial incentive (*R*)—two key policy instruments—jointly determine the private investment timing (τ), and to compare this outcome with the integrated benchmark.

### 2.2 A simple game between a health authority and a pharmaceutical firm

Consider a pharmaceutical firm (PF) that is allowed to invest in developing a drug. The investment is irreversible, entailing a sunk cost *I*>0 which is paid at the start of the project. We assume that an advance purchase commitments contract is signed between a health authority (HA) and the PF specifying: (1) the quality standard of the drug, and (2) the remuneration to the firm for an approved drug. For simplicity, the remuneration is assumed to be a constant and indefinite cash flow *R*. The amount *R* is treated as a parameter.^2^

Upon project completion, an independent adjudication committee (IAC) will determine whether the product meets the predefined technical specifications (2). The pre-specified quality standard is denoted by $q_{c}^{A}$ in the model. We simplify the model by not explicitly detailing the verification phase and assume that *q* is perfectly observable to both the PF and the HA. Similar to many R&D projects, investment lags are present: the initial sunk cost is incurred well before future revenue generation, reflecting the time required to achieve the specified quality standard.

Every pharmaceutical project can be conceptualized as a sequence of sub-projects, encompassing various stages such as discovery, pre-clinical, phase I, II, and III trials, and approval (18), (19). To focus on the specific issues of interest in this study, we simplify the project by merging the aforementioned stages into two major phases: the discovery phase before investment and the R&D phase after investment.

Uncertainty is modeled on a probability space (Ω, F, **P**). The firm's revenues depend on the quality of the drug, and the progression is captured through a geometric Brownian motion (GBM) (_*q*_*t*_)*t*≥0_. Before initiating the R&D process, the manufacturer will conduct basic research during the discovery phase, incurring lower costs to collect as much beneficial information as possible for the R&D process. Without loss of generality, we assume the cost of the discovery phase is 0. Nonetheless, the basic research is only expected to marginally enhance the quality of the product, as described by the stochastic differential equation (SDE)


$$\begin{array}{l} dq_{t}=\mu_{1}q_{t}dt+\sigma q_{t}dB_{t},\;q_{0}=q,\text{P}\text{-a.s.}, \end{array}$$ (1)

where *B*_*t*_ is a Wiener process. We take the natural filtration $\text{F}:={\left(F^{q}\right)}_{t\geq 0}$ on (Ω, F).

Once the PF considers the project sufficiently promising (to be determined endogenously), a sunk cost *I* will be paid, and the R&D phase starts. This will increase the expected growth rate of the drug's quality to μ_2_ (μ_2_>μ_1_>0). To summarize, if *T*≥0 is the time of investment, then the drug's quality evolves as


$$\begin{array}{l} dq_{t}=\{\begin{array}{cc} \mu_{1}q_{t}dt+\sigma q_{t}dB_{t} & \;if\;t<T,\\ \mu_{2}q_{t}dt+\sigma q_{t}dB_{t} & \;if\;t\geq T. \end{array} \end{array}$$ (2)

Now consider the following sequential game between the health authority and the firm. The HA moves first by specifying a quality standard $q_{c}^{A}$. After observing $q_{c}^{A}$, the PF chooses an optimal time τ to start the R&D project and pay the sunk cost. The firm stops further development of the product once $q_{c}^{A}$ is reached. Due to the stochastic nature of the evolution of *q*, the time τ takes the form of a *stopping time*.

### 2.3 The pharmaceutical firm's investment timing problem

The project timeline is as follows.


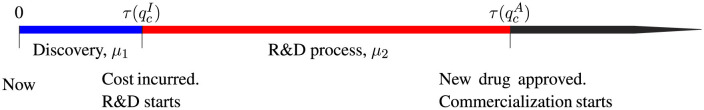


Upon achieving the quality standard $q_{c}^{A}$, the health authority will fulfill the advance purchase commitments by paying a constant flow *R* to the pharmaceutical company. The production cost of the drug, borne by the firm, is denoted by *C*. We assume that *C* is fixed and *R*>*C*. Furthermore, it is assumed that all decision makers involved are risk-neutral and share a common discount rate, ρ. The net present value (NPV) of commercialization at the quality level $q_{c}^{A}$ is calculated as follows.


$$\begin{array}{l} f\left(q_{c}^{A}\right)=\int_{0}^{\infty }e^{-\rho t}\left(R-C\right)dt=\frac{R-C}{\rho }. \end{array}$$ (3)

The firm's problem is to determine the optimal time ν to invest. After initiating the investment, the drug will be commercialized at the first hitting time of $q_{c}^{A}$, i.e., at


$$\begin{array}{l} \tau_{\nu }\left(q_{c}^{A}\right):=\inf\left\{t\geq \nu |q_{t}\geq q_{c}^{A}\right\}. \end{array}$$ (4)

The PF solves the optimal stopping problem


$$\begin{array}{l} V^{\mathrm{PF}}\left(q\right)=\sup_{\nu }{\text{E}}_{q}\left[\int_{\nu +\tau_{\nu }\left(q_{c}^{A}\right)}^{\infty }e^{-\rho t}\left(R-C\right)dt-e^{-\rho \nu }I\right]\\ =\sup_{\nu }{\text{E}}_{q}\left\{e^{-\rho \nu }{\text{E}}_{q_{\nu }}\left[e^{-\rho \tau_{\nu }\left(q_{c}^{A}\right)}{\text{E}}_{q_{c}^{A}}\left(\int_{0}^{\infty }e^{-\rho t}\left(R-C\right)dt\right)-I\right]\right\}\\ \equiv \sup_{\nu }{\text{E}}_{q}[e^{-\rho \nu }F\left(q_{\nu }\right)], \end{array}$$ (5)

where


$$\begin{array}{l} F\left(q_{\nu }\right)={\text{E}}_{q_{\nu }}\left[e^{-\rho \tau_{\nu }\left(q_{c}^{A}\right)}{\text{E}}_{q_{c}^{A}}(f\left(q_{c}^{A}\right))\right]-I={\text{E}}_{q_{\nu }}\left[e^{-\rho \tau_{\nu }\left(q_{c}^{A}\right)}f\left(q_{c}^{A}\right)\right]-I. \end{array}$$ (6)

The first line of Equation 5 shows the firm's trade-off: it chooses the investment time ν to maximize the expected discounted commercialization revenue (starting at the uncertain future time $\tau_{\nu }\left(q_{c}^{A}\right)$) when the standard is met), net of the sunk cost *I* paid at ν. The term $e^{-\rho \tau_{\nu }\left(q_{c}^{A}\right)}$ discounts the revenue back from the uncertain approval time to the investment time, and $e^{-\rho \tau_{\nu }}$ discounts the cost back to the present. The value function $V^{\mathrm{PF}}\left(q\right)$ thus represents the value of the firm's R&D option. The derivations of equations use the Markov property of the process (_*q*_*t*_)*t*≥0_. The last line reduces the problem to a standard optimal stopping problem. As in many problems of this kind, the optimal stopping time will take the form of the first hitting time of an endogenously determined investment trigger, which we will denote by $q_{c}^{I}$.^3^

To solve for this trigger, we first need to compute the value of *F*(*q*). Since $f\left(q_{c}^{A}\right)$ is a constant, it can be taken out of the expectation, and we obtain


$$\begin{array}{l} F\left(q\right)={\text{E}}_{q}\left[e^{-\rho \tau \left(q_{c}^{A}\right)}\right]f\left(q_{c}^{A}\right)-I. \end{array}$$ (7)

We now proceed to compute the *expected discount factor*, ${\text{E}}_{q}\left[e^{-\rho \tau \left(q_{c}^{A}\right)}\right]$. First, the *characteristic operator* of the process *q*_*t*_ after investment is


$$\begin{array}{l} L_{q}=\frac{1}{2}\sigma^{2}q^{2}\frac{\partial^{2}}{\partial q^{2}}+\mu_{2}q\frac{\partial }{\partial q}. \end{array}$$ (8)

Note that the general solution of the equation L_*q*_φ = ρφ is of the form,


$$\begin{array}{l} \varphi \left(q\right)=Aq^{\beta_{1}\left(\mu_{2}\right)}+Bq^{\beta_{2}\left(\mu_{2}\right)}, \end{array}$$ (9)

where β_1_(μ_2_)>1 and β_2_(μ_2_) < 0 are the two roots of the quadratic equation,


$$\begin{array}{l} Q_{2}\left(\beta \right)\equiv \frac{1}{2}\sigma^{2}\beta \left(\beta -1\right)+\mu_{2}\beta -\rho =0. \end{array}$$ (10)

We now think of the expected discount factor as a solution to this equation. Any such solution should solve the boundary condition φ(0) = 0, which can only be satisfied if *B* = 0. Therefore, ${\text{E}}_{q}\left[e^{-\rho \tau \left(q_{c}^{A}\right)}\right]=Aq^{\beta_{1}\left(\mu_{2}\right)}$, for some constant *A*, on $\left(q_{c}^{I},q_{c}^{A}\right)$.^4^

By using the Dynkin's formula,^5^ the expected discount factor can now easily be derived as


$$\begin{array}{l} {\text{E}}_{q_{c}^{I}}\left[e^{-\rho \tau \left(q_{c}^{A}\right)}\right]=\frac{\varphi \left(q_{c}^{I}\right)}{\varphi \left(q_{c}^{A}\right)}={\left(\frac{q_{c}^{I}}{q_{c}^{A}}\right)}^{\beta_{1}\left(\mu_{2}\right)}. \end{array}$$ (11)

So, the value of $F\left(q_{c}^{I}\right)$ is


$$\begin{array}{l} F\left(q_{c}^{I}\right)={\left(\frac{q_{c}^{I}}{q_{c}^{A}}\right)}^{\beta_{1}\left(\mu_{2}\right)}f\left(q_{c}^{A}\right)-I\\ \;={\left(\frac{q_{c}^{I}}{q_{c}^{A}}\right)}^{\beta_{1}\left(\mu_{2}\right)}\left(\frac{R-C}{\rho }\right)-I. \end{array}$$ (12)

By using the same approach above again, we can compute the expected discount factor for $q<q_{c}^{I}$:


$$\begin{array}{l} {\text{E}}_{q}\left[e^{-\rho \tau \left(q_{c}^{I}\right)}\right]={\left(\frac{q}{q_{c}^{I}}\right)}^{\beta_{1}\left(\mu_{1}\right)}, \end{array}$$ (13)

where β_1_(μ_1_)>1 is the positive root of the quadratic equation


$$\begin{array}{l} Q_{1}\left(\beta \right)\equiv \frac{1}{2}\sigma^{2}\beta \left(\beta -1\right)+\mu_{1}\beta -\rho =0. \end{array}$$ (14)

To utilize the results obtained, we can now establish the following proposition, which is proved in Appendix A.

Proposition 1. Suppose that the quality threshold $q_{c}^{A}$ is given. Then the value function of the R&D option for the pharmaceutical firm is given by


$$\begin{array}{l} V^{\mathrm{PF}}\left(q\right)=\\ \{\begin{array}{ll} {\left(\frac{q}{q_{c}^{I}}\right)}^{\beta_{1}\left(\mu_{1}\right)}\left[{\left(\frac{q_{c}^{I}}{q_{c}^{A}}\right)}^{\beta_{1}\left(\mu_{2}\right)}\left(\frac{R-C}{\rho }\right)-I\right] & \;if\;q<q_{c}^{I},\\ {\left(\frac{q}{q_{c}^{A}}\right)}^{\beta_{1}\left(\mu_{2}\right)}\left(\frac{R-C}{\rho }\right)-I & \;if\;q_{c}^{I}\leq q<q_{c}^{A},\\ \frac{R-C}{\rho } & \;if\;q\geq q_{c}^{A}. \end{array} \end{array}$$ (15)

Furthermore, the optimal investment threshold $q_{c}^{I}$ equals


$$\begin{array}{l} q_{c}^{I}=q_{c}^{A}{\left[\frac{\beta_{1}\left(\mu_{1}\right)}{\beta_{1}\left(\mu_{1}\right)-\beta_{1}\left(\mu_{2}\right)}\frac{\rho I}{R-C}\right]}^{1/\beta_{1}\left(\mu_{2}\right)}. \end{array}$$ (16)

From the pharmaceutical firm's perspective, the value of the project at any point in time is tied to the value of the stochastic process (_*q*_*t*_)*t*≥0_, which tracks the product's quality at that moment. If the product's quality is less than the optimal investment threshold $q_{c}^{I}$, the best strategy is to remain in the discovery phase, wait and observe the evolution of (_*q*_*t*_)*t*≥0_, and pay the sunk cost as soon as $q_{c}^{I}$ is reached. If the product's quality is larger than or equal to $q_{c}^{I}$, but less than the pre-specified quality $q_{c}^{A}$, the best strategy is to invest and commence the R&D process immediately. Finally, if the quality meets or surpasses $q_{c}^{A}$, there is no point in conducting basic research in the discovery phase and starting the R&D process; the best strategy of the pharmaceutical company is to promptly start the commercialization process by paying the operating cost *C* per period.

Proposition 1 provides the closed-form solution. The optimal investment threshold $q_{c}^{I}$ is increasing in the health authority's standard $q_{c}^{A}$, and the sunk cost *I*, and decreasing in the net revenue flow (*R*−*C*). The economic intuition is clear: a higher mandated standard increases the expected time and cost to reach commercialization after investment, raising the value of waiting during the discovery phase. This induces the firm to delay investment (higher $q_{c}^{I}$) until a higher-quality starting point is reached. Conversely, a more generous remuneration *R* increases the payoff, encouraging earlier investment (lower $q_{c}^{I}$), all else equal.

Note that so far we have implicitly assumed $q_{c}^{I}>0$. The following lemma, which is proved in Appendix B, shows that this is indeed the case.

** Lemma 1**. It holds that $\frac{\beta_{1}\left(\mu_{1}\right)}{\beta_{1}\left(\mu_{1}\right)-\beta_{1}\left(\mu_{2}\right)}>1$.

To ensure that proposition 1 makes economic sense, it is assumed that $\frac{\beta_{1}\left(\mu_{1}\right)}{\beta_{1}\left(\mu_{1}\right)-\beta_{1}\left(\mu_{2}\right)}<\frac{R-C}{\rho I}$, therefore, $q_{c}^{I}<q_{c}^{A}$. This condition will need to be satisfied so that the pharmaceutical has an incentive to start the project in the first place. Without meeting this condition, the firm will refrain from launching the R&D process, opting instead to prolong the discovery phase until the quality threshold is reached.

### 2.4 The health authority's standard-setting problem

In the previous subsection, we have computed the optimal investment threshold of the pharmaceutical firm, conditional on the quality threshold $q_{c}^{A}$ being given. For every potential quality standard established by the HA, there exists a unique corresponding optimal investment threshold for the PF. The objective of the HA is now to set an optimal quality standard $q_{c}^{A}$ to maximize the net present value of patients' health gain net of payments to the PF.

The HA thus solves a Stackelberg leader's problem: it anticipates the firm's reaction function $q_{c}^{I}\left(q_{c}^{A}\right)$ from Proposition 1, and chooses $q_{c}^{A}$ to maximize its own objective, which is the discounted stream of health benefits $Kq_{c}^{A}$ minus payments *R*, starting from the (endogenously determined) commercialization time.

For analytical convenience, we assume that patients' overall health outcomes are linearly correlated to the quality of the drug and that these outcomes can be expressed in monetary terms. In particular, the flow of health benefits is assumed to be fixed and equal to *K*>0. Hence, the problem of the health authority is given by


$$\begin{array}{l} V^{\mathrm{HA}}\left(q\right)=\\ \sup_{q_{c}^{A}}{\text{E}}_{q}\left[\int_{\tau \left(q_{c}^{I}\right)+\tau_{\tau \left(q_{c}^{I}\right)}\left(q_{c}^{A}\right)}^{\infty }e^{-\rho t}\left(Kq_{c}^{A}-R\right)dt\right]\\ =\sup_{q_{c}^{A}}{\text{E}}_{q}\left\{e^{-\rho \tau \left(q_{c}^{I}\right)}{\text{E}}_{q_{c}^{I}}\left[\int_{\tau_{\tau \left(q_{c}^{I}\right)}\left(q_{c}^{A}\right)}^{\infty }e^{-\rho t}\left(Kq_{c}^{A}-R\right)dt\right]\right\}\\ =\sup_{q_{c}^{A}}{\text{E}}_{q}\\ \left\{e^{-\rho \tau \left(q_{c}^{I}\right)}{\text{E}}_{q_{c}^{I}}\left[e^{-\rho \tau_{\tau \left(q_{c}^{I}\right)}\left(q_{c}^{A}\right)}{\text{E}}_{q_{c}^{A}}\left(\int_{0}^{\infty }e^{-\rho t}\left(Kq_{c}^{A}-R\right)dt\right)\right]\right\}\\ =\sup_{q_{c}^{A}}\left\{{\left(\frac{q}{q_{c}^{I}}\right)}^{\beta_{1}\left(\mu_{1}\right)}{\text{E}}_{q_{c}^{I}}[e^{-\rho \tau_{\tau \left(q_{c}^{I}\right)}\left(q_{c}^{A}\right)}v\left(q_{c}^{A}\right)]\right\}, \end{array}$$ (17)

where


$$\begin{array}{ll} v\left(q_{c}^{A}\right) & =\int_{0}^{\infty }e^{-\rho t}\left(Kq_{c}^{A}-R\right)dt=\frac{Kq_{c}^{A}-R}{\rho }. \end{array}$$ (18)

By using the same approach in the last subsection, we can compute the expected discount factor,


$$\begin{array}{l} {\text{E}}_{q_{c}^{I}}[e^{-\rho \tau_{\tau \left(q_{c}^{I}\right)}\left(q_{c}^{A}\right)}]={\left(\frac{q_{c}^{I}}{q_{c}^{A}}\right)}^{\beta_{1}\left(\mu_{2}\right)}. \end{array}$$ (19)

Similar to the problem of the firm, the issue facing the health authority can be reformulated as


$$\begin{array}{l} V^{\mathrm{HA}}\left(q\right)=\underset{q_{c}^{A}}{\sup}\left\{{\left(\frac{q}{q_{c}^{I}}\right)}^{\beta_{1}\left(\mu_{1}\right)}{\text{E}}_{q_{c}^{I}}[e^{-\rho \tau_{\tau \left(q_{c}^{I}\right)}\left(q_{c}^{A}\right)}]v\left(q_{c}^{A}\right)\right\}\\ \;=\underset{q_{c}^{A}}{\sup}\left\{{\left(\frac{q}{q_{c}^{I}}\right)}^{\beta_{1}\left(\mu_{1}\right)}{\left(\frac{q_{c}^{I}}{q_{c}^{A}}\right)}^{\beta_{1}\left(\mu_{2}\right)}v\left(q_{c}^{A}\right)\right\}. \end{array}$$ (20)

The subsequent proposition can be readily obtained.

Proposition 2. The value function of the health authority is


$$\begin{array}{l} V^{\mathrm{HA}}\left(q\right)=\\ \{\begin{array}{ll} {\left(\frac{q}{q_{c}^{I}}\right)}^{\beta_{1}\left(\mu_{1}\right)}\left[{\left(\frac{q_{c}^{I}}{q_{c}^{A}}\right)}^{\beta_{1}\left(\mu_{2}\right)}\left(\frac{Kq_{c}^{A}-R}{\rho }\right)\right] & \;if\;q<q_{c}^{I},\\ {\left(\frac{q}{q_{c}^{A}}\right)}^{\beta_{1}\left(\mu_{2}\right)}(\frac{Kq_{c}^{A}-R}{\rho }) & \;if\;q_{c}^{s}I\leq q<q_{c}^{A},\\ \frac{Kq-R}{\rho } & \;if\;q\geq q_{c}^{A}. \end{array} \end{array}$$ (21)

The value of the health authority's project depends on the current quality of the product. If the current quality $q<q_{c}^{I}$, the pharmaceutical company will stay in the discovery phase and wait until the optimal investment threshold is reached before proceeding with investment. After investment, the R&D process continues till the optimal quality standard is achieved, at which point the HA realizes its payoff. If the current quality $q_{c}^{I}\leq q<q_{c}^{A}$, the pharmaceutical company will start the R&D process immediately, and the payoff is realized when the optimal quality standard is met. Finally, if $q\geq q_{c}^{A}$, indicating the quality of the product has exceeded the optimal quality standard, the HA can promote product sales immediately.

Proposition 3. The optimal quality standard that maximizes the health authority's value is $q_{c}^{A}=\frac{\beta_{1}\left(\mu_{1}\right)}{\beta_{1}\left(\mu_{1}\right)-1}\frac{R}{K}$.Consequently, the firm's optimal investment threshold is


$$\begin{array}{l} q_{c}^{I}=\frac{\beta_{1}\left(\mu_{1}\right)}{\beta_{1}\left(\mu_{1}\right)-1}\frac{R}{K}{\left[\frac{\beta_{1}\left(\mu_{1}\right)}{\beta_{1}\left(\mu_{1}\right)-\beta_{1}\left(\mu_{2}\right)}\frac{\rho I}{R-C}\right]}^{1/\beta_{1}\left(\mu_{2}\right)}. \end{array}$$ (22)

The proof of this proposition can be found in Appendix C.

Proposition 3 reveals the HA's trade-off. A higher standard $q_{c}^{A}$ directly increases the per-period health benefit $Kq_{c}^{A}$ but, as in Proposition 1, indirectly delays commercialization by raising the firm's investment threshold $q_{c}^{I}$ and thus the expected time to approval. The optimal standard balances this benefit-delay trade-off. The solution shows $q_{c}^{A}$ is proportional to the remuneration-price ratio *R*/*K*. Intuitively, if the HA values health highly (large K) relative to its payment R, it sets a high standard. If the payment is high relative to the health valuation, it opts for a lower standard to accelerate access.

Since the HA's remuneration to the PF is a constant, the only decision that PF has to make revolves around the timing of its investment, irrespective of the timing of commercialization. In this case, once the quality standard $q_{c}^{A}$ is attained, it is optimal for the PF to commercialize the drug immediately. For the HA, the only decision lies in establishing the quality standard, designed to maximize patients' health gains net of payoffs to the firm, conditional on the reaction function of the PF.

## 3 The integrated social planner's problem (the benchmark)

In the decentralized framework, both the pharmaceutical firm and the health authority play pivotal roles, each overseeing a specific aspect of the project. The health authority, acting first, sets the commercialization approach by establishing a quality benchmark for the final product. Subsequently, based on this benchmark, the pharmaceutical firm decides on the optimal time to initiate the R&D phase, focusing on drug development. The primary objective for each party is to maximize the value of their segment of the project. This narrative shifts when introducing a social planner (SP) who consolidates control over both the R&D and commercialization facets. This unified approach necessitates a simultaneous consideration of the investment timing and the determination of an ideal quality standard. The social planner's mission diverges from the decentralized model by aiming to amplify the total value of the project, thereby harmonizing the strategies for development and market introduction under a single, overarching goal.

The optimal investment threshold and the optimal quality standard are denoted by $q_{I}^{I}$ and $q_{I}^{A}$, respectively, in the social planner's project. The problem of the social planner is


$$\begin{array}{l} V^{\mathrm{SP}}\left(q\right)=\underset{q_{I}^{A},q_{I}^{I}}{\sup}{\text{E}}_{q}\left[\int_{\tau \left(q_{I}^{A}\right)}^{\infty }e^{-\rho t}\left(Kq_{I}^{A}-C\right)dt-e^{-\rho \tau \left(q_{I}^{I}\right)}I\right]\\ \;=\underset{q_{I}^{A},q_{I}^{I}}{\sup}\left\{{\left(\frac{q}{q_{I}^{I}}\right)}^{\beta_{1}\left(\mu_{1}\right)}\left[{\left(\frac{q_{I}^{I}}{q_{I}^{A}}\right)}^{\beta_{1}\left(\mu_{2}\right)}\left(\frac{Kq_{I}^{A}-C}{\rho }\right)-I\right]\right\}. \end{array}$$ (23)

The social planner's value function $V^{\mathrm{SP}}\left(q\right)$ internalizes the entire net social benefit stream ($Kq_{I}^{A}-C$), avoiding the transfer payment *R*. The key distinction from the decentralized case is that both control variables, $q_{I}^{A}$ and $q_{I}^{I}$, are chosen simultaneously to maximize this unified objective.

The investment problem can be solved by treating $q_{I}^{A}$ as a constant and taking the partial derivatives of $V^{\mathrm{SP}}\left(q\right)$ with respect to $q_{I}^{I}$, and $\frac{\partial V^{\mathrm{SP}}\left(q\right)}{\partial q_{I}^{I}}=0$ gives


$$\begin{array}{l} q_{I}^{I}=q_{I}^{A}{\left(\frac{\beta_{1}\left(\mu_{1}\right)}{\beta_{1}\left(\mu_{1}\right)-\beta_{1}\left(\mu_{2}\right)}\frac{\rho I}{Kq_{I}^{A}-C}\right)}^{1/\beta_{1}\left(\mu_{2}\right)}. \end{array}$$ (24)

The problem of determining the optimal quality standard to enhance social welfare can be solved by taking the partial derivatives of $V^{\mathrm{SP}}\left(q\right)$ with respect to $q_{c}^{A}$, and $\frac{\partial V^{\mathrm{SP}}\left(q\right)}{\partial q_{c}^{A}}=0$ gives


$$\begin{array}{l} q_{I}^{A}=\frac{\beta_{1}\left(\mu_{2}\right)}{\beta_{1}\left(\mu_{2}\right)-1}\frac{C}{K}. \end{array}$$ (25)

Replacing $q_{I}^{A}$ in Equation 24, we have


$$\begin{array}{l} q_{I}^{I}=\frac{\beta_{1}\left(\mu_{2}\right)}{\beta_{1}\left(\mu_{2}\right)-1}\frac{C}{K}{\left[\frac{\beta_{1}\left(\mu_{1}\right)}{\beta_{1}\left(\mu_{1}\right)-\beta_{1}\left(\mu_{2}\right)}\left(\beta_{1}\left(\mu_{2}\right)-1\right)\frac{\rho I}{C}\right]}^{1/\beta_{1}\left(\mu_{2}\right)}. \end{array}$$ (26)

In addition, to ensure Equation 24 makes economic sense, it is assumed that $q_{I}^{I}\leq q_{I}^{A}$. Thus, the following condition must be satisfied.


$$\begin{array}{l} C>\frac{\beta_{1}\left(\mu_{1}\right)\left(\beta_{1}\left(\mu_{2}\right)-1\right)}{\beta_{1}\left(\mu_{1}\right)-\beta_{1}\left(\mu_{2}\right)}\rho I. \end{array}$$ (27)

Equations 24, 26 are the core results for the social planner. The optimal quality standard $q_{I}^{A}$ is proportional to the cost-to-benefit ratio (*C*/*K*), and crucially, is independent of the sunk cost *I*. This contrasts sharply with the decentralized standard $q_{c}^{A}$ (Proposition 3), which depends on the remuneration *R*. Here, the planner sets the standard based solely on the fundamental trade-off between the marginal cost of production (*C*) and the marginal social value of quality (*K*).

Proposition 4. The value of the project managed by the social planner $V^{\mathrm{SP}}\left(q\right)$ is maximized if the R&D process starts at $q_{I}^{I}$ and the quality standard is set to be $q_{I}^{A}$.

The proof of this proposition can be found in Appendix D.

It then immediately follows that

Proposition 5. the project value of the social planner is


$$\begin{array}{l} V^{\mathrm{SP}}\left(q\right)=\{\begin{array}{ll} {\left(\frac{q}{q_{I}^{I}}\right)}^{\beta_{1}\left(\mu_{1}\right)}\left[{\left(\frac{q_{I}^{I}}{q_{I}^{A}}\right)}^{\beta_{1}\left(\mu_{2}\right)}\left(\frac{Kq_{I}^{A}-C}{\rho }\right)\right] & \;if\;q<q_{I}^{I},\\ {\left(\frac{q}{q_{I}^{A}}\right)}^{\beta_{1}\left(\mu_{2}\right)}(\frac{Kq_{I}^{A}-C}{\rho }) & \;ifq_{I}^{I}\leq q<q_{I}^{A},\\ \frac{Kq-C}{\rho } & \;if\;q\geq q_{I}^{A}. \end{array} \end{array}$$ (28)

The project value of the social planner is dependent on the quality of the product. If the current quality is below the optimal investment threshold, i.e., $q<q_{I}^{I}$, the social planner will consider staying in the discovery phase, waiting, and collecting more information, and invest until the quality is high enough. If the current quality is higher than the optimal investment threshold but lower than the optimal quality standard, i.e., $q_{I}^{I}\leq q<q_{I}^{A}$, the social planner will skip the discovery phase and start the R&D process immediately. The R&D process will be completed when the optimal quality standard is first met. Finally, if the current quality is larger than the optimal quality standard, i.e., $q\geq q_{I}^{A}$, both the discovery phase and R&D process can be skipped. The best strategy is to proceed directly to commercialization.

## 4 Analysis of thresholds, project values, and practical implications

Throughout our analysis, we have identified four distinct thresholds for the two projects including (1) $q_{c}^{I}$, the optimal investment threshold of the PF, (2) $q_{c}^{A}$, the optimal quality standard set by the HA, (3) $q_{I}^{I}$, the optimal investment threshold set by the SP, and (4) $q_{I}^{A}$, the optimal quality standard set by the SP. To make the investment problems more interesting, we assume that the optimal investment thresholds are positioned below the optimal quality standards. Otherwise, achieving the quality standard during the discovery phase alone, without initiating the R&D process, would be an outcome that seldom aligns with the practical complexities and challenges encountered in real-world scenarios.

More specifically, in the decentralized project, we assume that $q_{c}^{I}<q_{c}^{A}$. The corresponding condition to be satisfied is


$$\begin{array}{l} R>\frac{\beta_{1}\left(\mu_{1}\right)}{\beta_{1}\left(\mu_{1}\right)-\beta_{1}\left(\mu_{2}\right)}\rho I+C. \end{array}$$ (29)

In the project conducted by the SP, we assume that $q_{I}^{I}<q_{I}^{A}$. The corresponding condition to be satisfied is


$$\begin{array}{l} C>\frac{\beta_{1}\left(\mu_{1}\right)\left(\beta_{1}\left(\mu_{2}\right)-1\right)}{\beta_{1}\left(\mu_{1}\right)-\beta_{1}\left(\mu_{2}\right)}\rho I. \end{array}$$ (30)

Next, we discuss the relationship between the two optimal investment thresholds.

Proposition 6. The investment threshold chosen by the SP is always strictly less than the one chosen by the PF, i.e., $q_{I}^{I}<q_{c}^{I}$.

The proof of this proposition can be found in Appendix E.

This result underscores a fundamental inefficiency of decentralization: strategic separation induces delay. The social planner, by internalizing the full health benefit of earlier availability, finds it optimal to trigger development sooner. In practical terms, this suggests that mission-driven, publicly coordinated R&D programs (which approximate the social planner) can be expected to initiate projects earlier than those relying solely on private firms reacting to regulatory and market signals. This is clearly relevant to time-sensitive health crises, such as pandemics, where accelerating the start of R&D is paramount.

**Figure 1:**
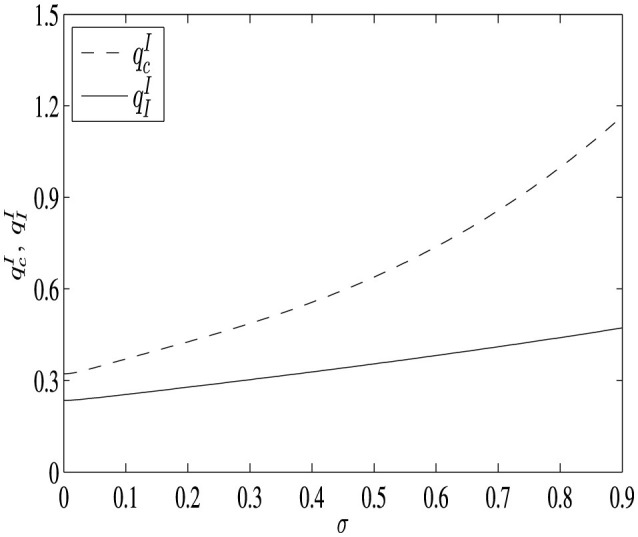
The difference of investment thresholds as uncertainty varies. The dashed line represents the investment threshold of the pharmaceutical firm, while the solid line indicates the investment threshold of the social planner.

To illustrate the distinction between the two investment thresholds more clearly, we consider a numerical example with the following parameter values: μ_1_ = 0.02, μ_2_ = 0.2, σ∈[0.001, 0.9], *I* = 10, ρ = 0.5, *C* = 20, *K* = 100, and *R* = 80. In Figure 1, we demonstrate that the investment threshold determined by the social planner is consistently lower. Given the identical growth rates μ_1_ and μ_2_, as well as the volatility σ for the stochastic process (_*q*_*t*_)*t*≥0_ in both projects, a lower investment threshold implies earlier investment and commencement of the R&D process.

The divergence in investment thresholds can be attributed to the differing organizational structures of the two projects. In the decentralized model, the objectives of the two parties are to maximize the value of their respective projects. From the perspective of the HA, for a certain quality standard, the sooner the investment takes place, the higher the payoffs. Earlier investments accelerate the development process by increasing growth rates, while simultaneously reducing the discount impact on the anticipated gains in patient health. However, from the perspective of the PF, earlier investments imply giving up information and the opportunity to improve product quality during the discovery phase. To capture option value in the discovery phase, the PF tends to wait and invest when the quality of the product is high enough.

Conversely, in the project managed by the SP, the objective is to maximize the project's overall value. Both the timing of investment and the quality standard are considered simultaneously. For the same quality standard, earlier investments lead to earlier realization of patients' health gains, which is consistent with the benefit of the social planner. Although earlier investments benefit the HA in the decentralized model, they do not necessarily benefit the PF. The conflict between the two players in the decentralized project leads to late investment. Hence, the investment threshold in the social planner's project is lower in the absence of conflicts.

A key distinction between the two projects is that the PF receives remuneration, *R*, from the HA upon successful completion of the R&D process, provided the drug meets the established quality threshold. However, in the project overseen by the SP, the parameter *R* is not a consideration. We will further examine how the level of remuneration influences the thresholds within the decentralized project and how *R* affects the determination of optimal quality standards across different projects.

For computational convenience, we denote *M* as the right hand side of the inequality (Equation 29), i.e., $M=\frac{\beta_{1}\left(\mu_{1}\right)}{\beta_{1}\left(\mu_{1}\right)-\beta_{1}\left(\mu_{2}\right)}\rho I+C$, and denote $N=\frac{\left(\beta_{1}\left(\mu_{1}\right)-1\right)\beta_{1}\left(\mu_{2}\right)}{\left(\beta_{1}\left(\mu_{2}\right)-1\right)\beta_{1}\left(\mu_{2}\right)}C$.

Proposition 7. Findings on remuneration *R* and quality standards. When $0<I<\frac{{\left(\beta_{1}\left(\mu_{1}\right)-\beta_{1}\left(\mu_{2}\right)\right)}^{2}C}{\beta_{1}{\left(\mu_{1}\right)}^{2}\left(\beta_{1}\left(\mu_{2}\right)-1\right)\rho }$, *M* < *N*. If *M* < *R* < *N*, the optimal quality standard of the decentralized project is less than the one managed by the social planner i.e., $q_{c}^{A}<q_{I}^{A}$. Hence, $q_{I}^{I}<q_{c}^{I}<q_{c}^{A}<q_{I}^{A}$.

The proof of this proposition can be found in Appendix F.

The optimal quality standard of the social planner $q_{I}^{A}$ is not affected by the variations of remuneration level since there is no remuneration in the social planner's project. However, a lower remuneration level will lead to a lower quality standard in the decentralized project $q_{c}^{A}$ (see Proposition 3). The sunk cost does not directly affect the quality standards of both projects. In the social planner's project, the decrease in sunk cost will lead to a lower required operating cost (see Equation 30), which will not affect the quality standard provided the operating cost remains sufficiently high. Similarly, the quality standard of the decentralized project $q_{c}^{A}$ is indirectly affected by sunk costs. A lower sunk cost will lead to a lower required remuneration (refer to Equation 29). However, as long as the minimum requirement of *R* is satisfied, i.e., if Equation 29 holds, the sunk cost *I* will not influence the quality standard of the decentralized project. The proposition shows that when *R* is low, the optimal quality standard for the decentralized project is lower than that managed by the social planner. Policy advice is that, if the budget for remuneration is limited while the project's sunk cost is relatively low, a social planner's project is preferred. This approach not only ensures an earlier start and, consequently, quicker completion and commercialization of the product, but also guarantees a higher quality standard.

Proposition 8. Findings on Remuneration *R* and Quality Standards. When $0<I<\frac{{\left(\beta_{1}\left(\mu_{1}\right)-\beta_{1}\left(\mu_{2}\right)\right)}^{2}C}{\beta_{1}{\left(\mu_{1}\right)}^{2}\left(\beta_{1}\left(\mu_{2}\right)-1\right)\rho }$, *M*<*N* and when $I\geq \frac{{\left(\beta_{1}\left(\mu_{1}\right)-\beta_{1}\left(\mu_{2}\right)\right)}^{2}C}{\beta_{1}{\left(\mu_{1}\right)}^{2}\left(\beta_{1}\left(\mu_{2}\right)-1\right)\rho }$, *M*≥*N*. If *R*≥max{*M, N*}, the optimal quality standard of the project managed by the social planner is less or equal to that of the decentralized project, i.e., $q_{I}^{A}\leq q_{c}^{A}$. Moreover, there is a unique remuneration level *R*_*c*_ (*R*_*cc*_) such that the optimal investment threshold of the decentralized project equals the optimal quality standard of the social planner's project, i.e., $q_{c}^{I}=q_{I}^{A}$. Thus if *N*(*M*) ≤ *R*<*R*_*c*_(*R*_*cc*_), $q_{I}^{I}<q_{c}^{I}<q_{I}^{A}<q_{c}^{A}$. If *R*≥*R*_*c*_(*R*_*cc*_), $q_{I}^{I}<q_{I}^{A}\leq q_{c}^{I}<q_{c}^{A}$.

The proof of this proposition can be found in Appendix G.

Increasing the remuneration level can reverse the result in Proposition 7. When *R*≥max{*M, N*}, the quality standard of the decentralized project $q_{c}^{A}$ increases while the quality standard of the social planner's project $q_{I}^{A}$ is not affected. Moreover, as *R* increases, the investment threshold of the decentralized project $q_{c}^{I}$ first decreases and then increases. When *N*(*M*) ≤ *R*<*R*_*c*_(*R*_*cc*_), the investment threshold of the decentralized project is less than the quality standard of the social planner's project, i.e., $q_{c}^{I}<q_{I}^{A}$. If *R* further increases, when *R*≥*R*_*c*_(*R*_*cc*_), the investment threshold of the decentralized project will be larger or equal to the quality standard of the social planner's project, i.e., $q_{c}^{I}\geq q_{I}^{A}$.

With sufficient funding, the remuneration level *R* can be used as a tool to adjust the product's quality standard in the decentralized project, which is not possible in the social planner's project. However, this comes at the cost of delaying commercialization later, since $q_{c}^{I}$ also increases with *R*. In an extreme case, for instance, when *R* takes values in *R*≥*R*_*c*_(*R*_*cc*_), it can be the case that the final product in the social planner's project has been finished while the R&D process has not even started yet in the decentralized project. Moreover, it can be proved that *R*_*cc*_ ≤ *R*_*c*_. In other words, the higher the sunk cost *I*, the less remuneration *R* is required to make $q_{c}^{I}$ approach $q_{I}^{A}$ from below. Mathematically, this is because $q_{c}^{I}$ increases with *I* while $q_{I}^{A}$ is not affected if the operating cost *C* reaches the minimum value, i.e., $C>\frac{\beta_{1}\left(\mu_{1}\right)\left(\beta_{1}\left(\mu_{2}\right)-1\right)}{\beta_{1}\left(\mu_{1}\right)-\beta_{1}\left(\mu_{2}\right)}\rho I$.

In summary, the central policy insight from Propositions 7, 8 is the explicit trade-off between quality ambition and development speed that a payer/regulator faces in a decentralized system. This directly models a key dilemma in designing pull incentives like APCs: setting a high reward to attract investment toward a high-quality target may perversely slow down the very innovation it seeks to spur, if the associated standard is set too high. This mirrors ongoing debates about balancing the encouragement of innovation with rapid access to new technologies.

**Figure 2:**
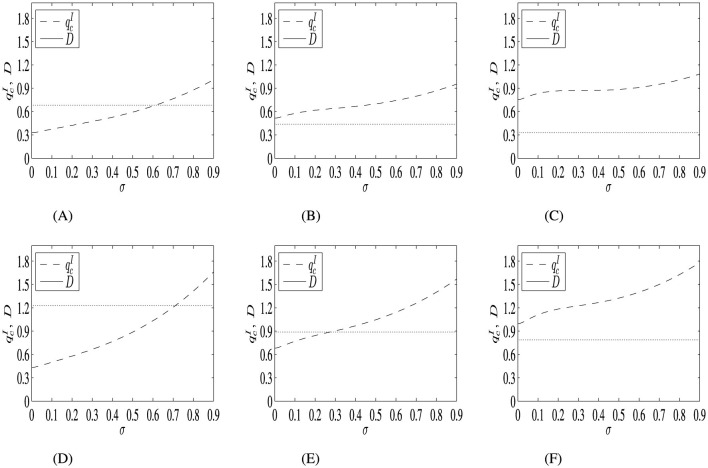
The investment threshold of the decentralized project varies with uncertainty, and the range between the maximum and minimum investment threshold values shifts under different sunk costs *I* and remuneration level *R*. The parameter values are: μ_1_ = 0.02, μ_2_ = 0.2, σ = [0.001, 0.9], *I* = 0.5 or 1, ρ = 0.5, *C* = 5, *K* = 10, and *R*=10, 30 or 60. In the figures above, *D* represents the difference between the maximum and minimum investment threshold. **(A)** *I* = 0.5, *R* = 10. **(B)** *I* = 0.5, *R* = 30. **(C)** *I* = 0.5, *R* = 60. **(D)** *I* = 1, *R* = 10. **(E)** *I* = 1, *R* = 30. **(F)** *I* = 1, *R* = 60.

In Figure 2, we analyze how the investment threshold of the decentralized project varies with uncertainty, and how the spread of the maximum and minimum value of the threshold changes under different sunk costs and levels of remuneration.

The following proposition is easily obtained.

Proposition 9. The spread between the maximum and minimum values of the investment threshold narrows as the remuneration level *R* rises, under varying degrees of uncertainty. Conversely, the spread widens as the sunk cost *I* increases.

The investment threshold of the decentralized project increases with uncertainty. Moreover, as the remuneration level *R* increases, the spread between the maximum and minimum values of the investment threshold decreases. The effect of *R* on the investment threshold $q_{c}^{I}$ can be separated into two parts. From Proposition 3, we know that $q_{c}^{I}=\frac{\beta_{1}\left(\mu_{1}\right)}{\beta_{1}\left(\mu_{1}\right)-1}\frac{R}{K}{\left[\frac{\beta_{1}\left(\mu_{1}\right)}{\beta_{1}\left(\mu_{1}\right)-\beta_{1}\left(\mu_{2}\right)}\frac{\rho I}{R-C}\right]}^{1/\beta_{1}\left(\mu_{2}\right)}$ which can be considered as a product of two terms. First, an increase in *R* will lead to a higher quality standard since $q_{c}^{A}=\frac{\beta_{1}\left(\mu_{1}\right)}{\beta_{1}\left(\mu_{1}\right)-1}\frac{R}{K}$. This is quite intuitive since a product with higher quality often requires more inputs of the available resources, such as time, money, and effort. To increase incentives to consume more resources in the research and development of a product, higher remuneration will be needed. The impact of the remuneration level *R* on the optimal quality standard $q_{c}^{A}$ is termed the “quality effect.” This effect typically leads to a postponement of the investment decision. On the other hand, an elevation in *R* signals higher anticipated future revenues, prompting the decision maker to prefer accessing these revenues at an earlier stage. The impact of *R* on the project's Net Present Value (NPV) is referred to as the “NPV effect,” which serves as an incentive to advance the investment timing. This dynamic is encapsulated within the term ${\left[\frac{\beta_{1}\left(\mu_{1}\right)}{\beta_{1}\left(\mu_{1}\right)-\beta_{1}\left(\mu_{2}\right)}\frac{\rho I}{R-C}\right]}^{1/\beta_{1}\left(\mu_{2}\right)}$.

Figure 2 shows that when uncertainty is low, the investment threshold goes up faster when *R* increases. Intuitively, as *R* increases, the quality standard increases. If uncertainty is low, the chances of large upward jumps are lower. In this case, a better strategy is to wait in the discovery phase and invest after the upward jumps are realized, rather than investing earlier and hoping that the upward jumps will occur during the R&D process, which would lead to a sooner finish of the project. Earlier investment means giving up the upward jumps for free, especially when these jumps are more valuable when uncertainty is low, since they happen less often. Since the upward jumps are more valuable when uncertainty is low, it is more important to capture them by investing later when the current quality is higher, which is represented by a higher optimal investment threshold. In other words, the “quality effect” is larger when *R* goes up with lower uncertainty. On the other hand, although the “NPV effect” tends to encourage earlier investment, it is limited by the “quality effect.” Sooner investment reduces the project's value when uncertainty is low, since valuable upward jumps are forgone. The stronger “quality effect” when uncertainty is low causes the optimal investment threshold to increase more rapidly.

When uncertainty is high, the “quality effect” is weaker since even if earlier investment means giving up the upward jumps in the discovery phase for free, it is more likely that these jumps will happen in the R&D process. Hence, it is less important to ensure that the benefits of upward jumps are captured before investment. And these jumps are less valuable because they occur more often. Moreover, “NPV effect” is stronger since an earlier investment is a possibility. The combination of the two effects leads to a slower increase in the threshold as *R* increases with higher uncertainty.

Since the “quality effect,” which leads to the increase of the investment threshold, is much stronger when uncertainty is low, while the “NPV effect,” which reduces the investment threshold, becomes stronger when uncertainty is higher, the spread of the maximum and minimum value of the investment threshold decreases as the remuneration level *R* increases. Moreover, the spread is also related to the value of sunk cost. The higher the sunk cost, the higher the spread. This is because the “NPV effect” is even weaker when *I* is large, while the “quality effect” is not affected. The combination of the two effects increases the spread.

To summarize, the finding of Proposition 9 is that the investment threshold's sensitivity to uncertainty varies with the level of remuneration R, offering nuanced guidance for risk-sharing in innovation contracts. In high-uncertainty projects (e.g., novel therapeutic platforms), a high R is more effective at accelerating investment because the “NPV effect” dominates. This supports the use of substantial milestone payments or guaranteed volumes in areas like antimicrobial or cancer vaccine development, where technical risk is high. Conversely, for lower-uncertainty projects, financial rewards are more likely to be channeled into pursuing higher quality, warranting closer payer oversight of standard-setting.

Next, we compare the value of the decentralized project to that of the social planner's project.

Proposition 10. The value of the project managed by the social planner exceeds than that of the decentralized one.

The proof of this proposition can be found in Appendix H.

**Figure 3:**
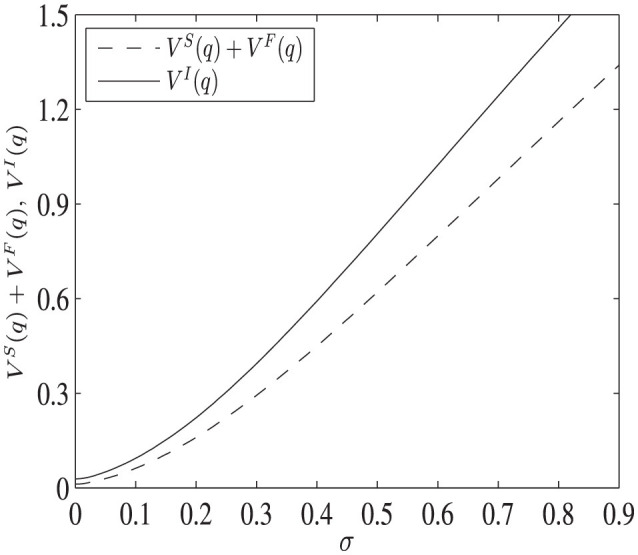
The difference of project values as uncertainty varies, when $q<q_{I}^{I}<q_{c}^{I}$. The dashed line represents the total value of PF and HA in the decentralized project, while the solid line shows the project's value managed by SP.

Two examples are provided to show the difference in the project values. Figure 3 shows the different project values when the initial value of the stochastic process *q* is smaller than the investment thresholds of both projects, i.e., $q<q_{I}^{I}<q_{c}^{I}$. The parameter values are: *q* = 0.3, μ_1_ = 0.02, μ_2_ = 0.2, σ = [0.001, 0.9], *I* = 0.5, ρ = 0.5, *C* = 5, *K* = 10, and *R* = 7. In this case, the best strategy for both decision makers is to wait until each investment threshold is reached before paying the sunk cost. It is shown that the value of the social planner's project is strictly larger than that of the decentralized one.

Intuitively, although optimal decisions are made in both projects, the maximization problem that $q_{I}^{I}$ solves is an unconstrained one, while $q_{c}^{I}$ solves a constrained maximization problem, in which the pharmaceutical company moves next for a given quality standard set by the government in advance. In this case, the option value of the pharmaceutical company's project cannot be fully captured, resulting in a lower total value for the decentralized project. Furthermore, as uncertainty σ increases, the value of the decentralized project increases more slowly, indicating that part of the option value is not captured, since the decision of the pharmaceutical company is constrained by the action of the health authority.

**Figure 4:**
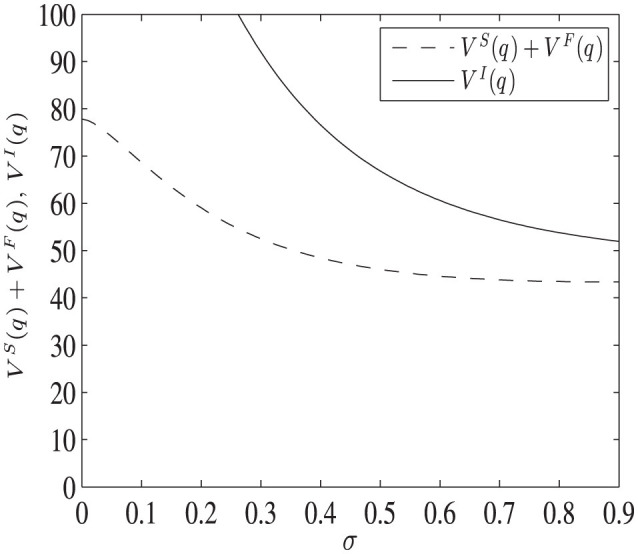
The difference of project values as uncertainty varies, when $q_{I}^{I}<q_{c}^{I}<q$. The dashed line represents the total value of PF and HA in the decentralized project, while the solid line shows the project's value managed by SP.

Figure 4 shows the different project values when the start value of the stochastic process *q* is larger than the investment thresholds of both projects, i.e., $q_{I}^{I}<q_{c}^{I}<q$. The parameter values are: *q* = 2, μ_1_ = 0.02, μ_2_ = 0.2, σ = [0.001, 0.9], *I* = 1, ρ = 0.5, *C* = 5, *K* = 10, *R* = 20. In this case, both projects will start immediately without further waiting, and it is shown that the social planner's project value is strictly larger than that of the decentralized one.

In this case, the decision made by the government is also affected by the response of the pharmaceutical company in the decentralized project. In other words, the health authority, although it moves first by setting a quality standard, also solves a constrained maximization problem, restricted by what the pharmaceutical company will react to, given the set quality standard. Thus, the optimal investment threshold $q_{c}^{A}$ does not maximize the unconstrained value function *x*↦*NPV*(*x*) (see Appendix H). Hence, the total value of the decentralized project is less than the social planner's project. In addition, as uncertainty goes up, both project values decrease. This is because both projects have no flexibility but to invest immediately. Without the option to wait, the bad outcomes cannot be avoided, and increasing uncertainty reduces the value of both projects.

This fundamental welfare result quantifies the cost of fragmented decision-making. The combined value of the HA and PF is less than the value a coordinated planner could achieve. In policy terms, this efficiency loss justifies exploring hybrid models that better align incentives, such as: (1) Public-private partnerships (PPPs) with integrated governance, which move closer to the social planner benchmark; (2) Regulatory-design partnerships where developers and regulators collaborate early on defining feasible yet meaningful development endpoints (akin to co-setting $q_{c}^{A}$); and (3) Value-based agreements that more tightly link the level of payment *R* to the achieved health outcomes, partially internalizing the planner's objective.

## 5 Conclusions and policy implications

This study provides a formal analysis of how a health authority's quality standard affects a pharmaceutical firm's R&D investment timing, comparing decentralized decision-making with an integrated social planner benchmark. Our findings, grounded in real options theory, offer specific insights into designing innovation incentives in healthcare. The main conclusions and their policy implications are summarized as follows:

First, integrated coordination accelerates development but requires new governance models. Thus, for public health priorities where time is critical (e.g., pandemic preparedness, outbreaks of neglected diseases), there is a strong efficiency case for coordinated, public-sector-led or heavily orchestrated R&D programs that approximate the role of a social planner. This supports the creation of entities with the mandate and resources to manage both technical and incentive alignment, moving beyond simple funding contracts.

Second, remuneration and quality standards are dual policy levers with an inherent trade-off. Health authorities that introduce pull incentives, such as APCs or price-based pricing, are expected to address these trade-offs appropriately. Contracts should target clear minimum viable efficacy levels and strong financial incentives to ensure quick launch. For therapeutic area development where high quality is desired, larger rewards can be linked to correlated levels of efficacy.

Third, project characteristics should inform the choice of incentive framework. For low-budget, high-urgency applications, integrated models or tightly constrained PPPs can be adopted. For large-scale efforts to achieve breakthrough quality, decentralized models with carefully-tuned R and $q_{c}^{A}$ can be effective. High technical uncertainty calls for incentive structures that greatly reward success (high R) to overcome risk aversion, possibly based on success-dependent royalty rates or tiered pricing.

Finally, the measured efficiency loss justifies investing in alignment mechanisms. The welfare superiority of the social planner's outcome quantifies the efficiency loss from separated optimization. This loss represents the potential value to be gained from improved alignment mechanisms. Investments should be made in progressive regulatory pathways (e.g., breakthrough therapy designations, rolling reviews), early scientific advice to align on standards, and outcome-based payment models that share risk and better link rewards to societal value. Recent moves toward regulatory agility and managed-entry agreements in various health systems are practical steps in this direction.

In conclusion, our model reframes the challenge of stimulating pharmaceutical R&D as a problem of optimally coordinating two key decisions: how good and when to start. There is no general solution because the choice between decentralized and integrated strategies depends first on policy goals, resources, and disease context. By modeling the tradeoff between quality, financial rewards, and timing, we provide insights into the design of more effective, context-aware innovation policies.

## Data Availability

The original contributions presented in the study are included in the article/Supplementary material, further inquiries can be directed to the corresponding author.

## Footnotes

1 The END (Ending Neglected Diseases) fund. https://endfund.org/impact-stories/working-to-end-neglected-tropical-diseases-in-our-lifetimes/.

2 In practice, cash flows are typically paid over a certain period of time. However, for analysis, we assume these cash flows to be infinite. This simplification allows us to calculate the expected discounted value of the cash flows over a finite horizon by simply multiplying the value of the infinite cash flows by a discount factor. This approach facilitates easier application and analysis.

3 See Peskir and Shiryaev (20).

4 The inequality $q_{c}^{I}<q_{c}^{A}$ is, for now, an assumption. It will be verified later.

5 See Øksendal (21).
