# Supplementary material for: On quality standards and the timing of pharmaceutical investment
Source: Front Public Health. 2026 Jan 21;13:1738084. doi: 10.3389/fpubh.2025.1738084 (PMC12868235; doi:10.3389/fpubh.2025.1738084)
Supplement: Supplementary file 1 [file Data_Sheet_1.pdf]

Supplementary Material

APPENDIX

A PROOF OF PROPOSITION 1

The optimal quality threshold can be computed by taking the first order partial derivatives of eq. (15) with respect to q_c^I when $q \leq q_c^I$

$$\begin{aligned}\frac{\partial V^{\mathcal{PF}}(q)}{\partial q_c^I} =& -\beta_1(\mu_1)q^{\beta_1(\mu_1)}(q_c^I)^{-\beta_1(\mu_1)-1}\left[\left(\frac{q_c^I}{q_c^A}\right)^{\beta_1(\mu_2)}\left(\frac{R-C}{\rho}\right)-I\right]\\&+q^{\beta_1(\mu_1)}(q_c^I)^{-\beta_1(\mu_1)}\left[\beta_1(\mu_2)(q_c^I)^{\beta_1(\mu_2)}\left(\frac{R-C}{\rho}\right)\right],\end{aligned}$$

and $\frac{\partial V^{\mathcal{PF}}(q)}{\partial q_c^I} = 0$ gives

$$q_c^I = q_c^A\left[\frac{\beta_1(\mu_1)}{\beta_1(\mu_1)-\beta_1(\mu_2)}\frac{\rho I}{R-C}\right]^{1/\beta_1(\mu_2)}.$$

The second order partial derivatives of eq. (15) with respect to q_c^I is

$$\begin{aligned}\frac{\partial^2 V^{\mathcal{PF}}(q)}{\partial {q_c^I}^2} =& q^{\beta_1(\mu_1)}(q_c^I)^{-\beta_1(\mu_1)-2}\Bigg[\beta_1(\mu_1)(\beta_1(\mu_1)\\&-\beta_1(\mu_2))\left(\frac{q_c^I}{q_c^A}\right)^{\beta_1(\mu_2)}\left(\frac{R-C}{\rho}\right)-\beta_1(\mu_2)(\beta_1(\mu_2)+1)I\Bigg].\end{aligned}$$

Substituting $\left(\frac{q_c^I}{q_c^A}\right)^{\beta_1(\mu_2)}$ with $\frac{\beta_1(\mu_1)}{\beta_1(\mu_1)-\beta_1(\mu_2)}\frac{\rho I}{R-C}$,

$$\begin{aligned}&\frac{\partial^2 V^{\mathcal{PF}}(q)}{\partial {q_c^I}^2}\\&=q^{\beta_1(\mu_1)}(q_c^I)^{-\beta_1(\mu_1)-2}\Bigg[\beta_1(\mu_1)(\beta_1(\mu_1)-\beta_1(\mu_2))\frac{\beta_1(\mu_1)}{\beta_1(\mu_1)-\beta_1(\mu_2)}\frac{\rho I}{R-C}\frac{R-C}{\rho}\\&\quad-(\beta_1(\mu_1)+1)\beta_1(\mu_1)I\Bigg]\\&=q^{\beta_1(\mu_1)}(q_c^I)^{-\beta_1(\mu_1)-2}\Big[-\beta_1(\mu_1)I\Big].\end{aligned}$$

Since $q^{\beta_1(\mu_1)} > 0$ and $(q_c^I)^{-\beta_1(\mu_1)-2} > 0$, $\frac{\partial^2 V^{\mathcal{PF}}(q)}{\partial {q_c^I}^2} < 0$. q_c^I is the the optimal investment threshold that maximizes the pharmaceutical company's project value.

B PROOF OF LEMMA 1

We start by looking at the quadratic equation:

$$\mathbb{Q}(\beta) = \frac{1}{2}\sigma^2\beta(\beta - 1) + \mu\beta - \rho = 0.$$

$\beta_1 > 0$ is one of the two roots of the above equation.

Implicit differentiation of $\mathbb{Q}(\beta_1) = 0$ with respect to μ gives

$$\frac{\partial\mathbb{Q}(\beta_1)}{\partial\beta_1}\frac{\partial\beta_1}{\partial\mu} + \frac{\partial\mathbb{Q}(\beta_1)}{\partial\mu} = 0 \Leftrightarrow \frac{\partial\beta_1}{\partial\mu} = -\frac{\partial\mathbb{Q}(\beta_1)/\partial\mu}{\partial\mathbb{Q}(\beta_1)/\partial\beta_1}.$$

The denominator can be computed easily since the graph of $\mathbb{Q}(\beta)$ is a U-shape parabola and $\partial\mathbb{Q}(\beta_1)/\partial\beta_1 > 0$.

From the direct derivatives

$$\frac{\partial\mathbb{Q}(\beta_1)}{\partial\mu} = \beta_1 > 0.$$

So it follows that

$$\frac{\partial\beta_1}{\partial\mu} < 0.$$

Since we assume that $\mu_1 < \mu_2$, $\beta_1(\mu_1) > \beta_1(\mu_2)$, so

$$\frac{\beta_1(\mu_1)}{\beta_1(\mu_1) - \beta_1(\mu_2)} = 1 + \frac{\beta_1(\mu_2)}{\beta_1(\mu_1) - \beta_1(\mu_2)}.$$

Since $\beta_1(\mu_1) > \beta_1(\mu_2)$, $\beta_1(\mu_1) - \beta_1(\mu_2) > 0$,

$$\frac{\beta_1(\mu_1)}{\beta_1(\mu_1) - \beta_1(\mu_2)} > 1.$$

C PROOF OF PROPOSITION 3

The optimal quality threshold can be computed by taking the first order partial derivatives of the value function in Proposition 2, when $q < q_c^I$, with respect to q_c^A, and substituting q_c^I with $q_c^I = q_c^A \left[\frac{\beta_1(\mu_1)}{\beta_1(\mu_1)-\beta_1(\mu_2)}\frac{\rho I}{R-C}\right]^{1/\beta_1(\mu_2)}$ as it is in Proposition 1,

$$\begin{aligned}\frac{\partial V^{\mathcal{HA}}(q)}{\partial q_c^A} =& q^{\beta_1(\mu_2)}\left[\frac{\beta_1(\mu_1)}{\beta_1(\mu_1) - \beta_1(\mu_2)}\frac{\rho I}{R - C}\right]^{\frac{\beta_1(\mu_2)-\beta_1(\mu_1)}{\beta_1(\mu_2)}} \\ &\times\left[- \beta_1(\mu_2)(q_c^A)^{-\beta_1(\mu_2)-1}\left(\frac{Kq_c^A - R}{\rho}\right) + (q_c^A)^{-\beta_1(\mu_2)}\frac{K}{\rho}\right].\end{aligned}$$

For computational convenience, we denote

$$M = q^{\beta_1(\mu_2)} \left[\frac{\beta_1(\mu_1)}{\beta_1(\mu_1) - \beta_1(\mu_2)} \frac{\rho I}{R - C} \right]^{\frac{\beta_1(\mu_2) - \beta_1(\mu_1)}{\beta_1(\mu_2)}}.$$

So $\frac{\partial V^{\mathcal{HA}}(q)}{\partial q_c^A}$ can be rewritten as

$$\frac{\partial V^{\mathcal{HA}}(q)}{\partial q_c^A} = M \left[-\beta_1(\mu_2)(q_c^A)^{-\beta_1(\mu_2)-1} \left(\frac{Kq_c^A - R}{\rho} \right) + (q_c^A)^{-\beta_1(\mu_2)} \frac{K}{\rho} \right],$$

and $\frac{\partial V^{\mathcal{HA}}(q)}{\partial q_c^A} = 0$ gives

$$q_c^A = \frac{\beta_1(\mu_1)}{\beta_1(\mu_1) - 1} \frac{R}{K}.$$

The second order partial derivatives of eq. (20) with respect to q_c^A is

$$\frac{\partial^2 V^{\mathcal{HA}}(q)}{\partial {q_c^A}^2} = M(q_c^A)^{-\beta_1(\mu_2)-2} \left[\beta_1(\mu_2)(\beta_1(\mu_2)+1) \frac{Kq_c^A - R}{\rho} - \frac{2Kq_c^A}{\rho} \beta_1(\mu_2) \right].$$

Since $q_c^A = \frac{\beta_1(\mu_2)}{\beta_1(\mu_2)-1} \left(\frac{C}{K} \right)$,

$$\begin{aligned}
&\beta_1(\mu_2)(\beta_1(\mu_2)+1) \frac{Kq_c^A - R}{\rho} - \frac{2Kq_c^A}{\rho} \beta_1(\mu_2) \\
=&\beta_1(\mu_2)(\beta_1(\mu_2)-1) \frac{Kq_c^A}{\rho} - \beta_1(\mu_2)(\beta_1(\mu_2)+1) \frac{R}{\rho} \\
=&\beta_1(\mu_2)(\beta_1(\mu_2)-1) \frac{K}{\rho} \frac{\beta_1(\mu_2)}{\beta_1(\mu_2)-1} \frac{R}{K} - \beta_1(\mu_2)(\beta_1(\mu_2)+1) \frac{R}{\rho} \\
=&-\frac{R}{\rho} \beta_1(\mu_2) < 0.
\end{aligned}$$

Because M and q_c^A are both positive, $\frac{\partial^2 V^F(q)}{\partial^2 q_c^A} < 0$.

And it can also be proved that q_c^A maximizes the value function shown in the Proposition **??**, when $q_c^I \le q < q_c^A$.

$$\frac{\partial V^{\mathcal{HA}}(q)}{\partial q_c^A} = (-\beta_1(\mu_2)) \left(\frac{q}{q_A^c} \right)^{\beta_1(\mu_2)} \left(\frac{1}{q_c^A} \right) \left(\frac{Kq_c^A - R}{\rho} \right) + \left(\frac{q}{q_A^c} \right)^{\beta_1(\mu_2)} \frac{K}{\rho}$$

and $\frac{\partial V^{\mathcal{HA}}(q)}{\partial q_c^A} = 0$ gives

$$q_c^A = \frac{\beta_1(\mu_1)}{\beta_1(\mu_1) - 1} \frac{R}{K}.$$

The first order condition then becomes,

$$\frac{\partial V^{\mathcal{HA}}(q)}{\partial q_c^A} = \left(\frac{q(\beta_1(\mu_2) - 1)K}{\beta_1(\mu_2)R} \right)^{\beta_1(\mu_2)} \left(\frac{R}{(\beta_1(\mu_2) - 1)\rho} \right) > 0.$$

The second order condition is,

$$\begin{aligned} \frac{\partial^2 V^{\mathcal{HA}}(q)}{\partial {q_c^A}^2} =& (-\beta_1(\mu_2)) \left(\frac{q}{q_c^A} \right)^{\beta_1(\mu_2)} \left(\frac{1}{q_c^A} \right) \left[(1 - \beta_1(\mu_2)) \frac{K}{\rho} + \frac{\beta_1(\mu_2)R}{q_A^c \rho} \right] \\ &+ \left(\frac{q}{q_c^A} \right)^{\beta_1(\mu_2)} \left(- \frac{1}{(q_c^A)^2} \frac{\beta_1(\mu_2)R}{\rho} \right). \end{aligned}$$

When $q_c^A = \frac{\beta_1(\mu_1)}{\beta_1(\mu_1)-1} \frac{R}{K}$,

$$\frac{\partial^2 V^{\mathcal{HA}}(q)}{\partial {q_c^A}^2} = \left(\frac{q}{q_c^A} \right)^{\beta_1(\mu_2)} \left(- \frac{\beta_1(\mu_2)R}{(\beta_1(\mu_2) - 1)K} \right) < 0$$

Thus q_c^A is the optimal quality standard which maximizes the value of the health authority's project.

D PROOF OF PROPOSITION 4

The partial derivatives of $V^{\mathcal{SP}}(q)$ in eq. (23) with respect to q_I^I is

$$\begin{aligned} \frac{\partial V^{\mathcal{SP}}(q)}{\partial q_I^I} =& q^{\beta_1(\mu_1)} (q_I^I)^{\beta_1(\mu_2) - \beta_1(\mu_1) - 1} (q_I^A)^{-\beta_1(\mu_2)} \left(\frac{K q_I^A - C}{\rho} \right) (\beta_1(\mu_2) - \beta_1(\mu_1)) \\ &+ q^{\beta_1(\mu_1)} (q_I^I)^{-\beta_1(\mu_1) - 1} \beta_1(\mu_1) I, \end{aligned}$$

and $\frac{\partial V^{\mathcal{SP}}(q)}{\partial q_I^I} = 0$ gives $q_I^I = q_I^A \left(\frac{\beta_1(\mu_1) I}{\beta_1(\mu_1) - \beta_1(\mu_2)} \frac{\rho}{K q_I^A - C} \right)^{1/\beta_1(\mu_1)}$.

The partial derivatives of $V^{\mathcal{SP}}(q)$ with respect to q_I^A is

$$\begin{aligned} \frac{\partial V^{\mathcal{SP}}(q)}{\partial q_I^A} =& q^{\beta_1(\mu_1)} (q_I^I)^{\beta_1(\mu_2) - \beta_1(\mu_1)} \\ & \times \left[-\beta_1(\mu_2) (q_I^A)^{-\beta_1(\mu_2) - 1} \left(\frac{K q_I^A - C}{\rho} \right) + (q_I^A)^{-\beta_1(\mu_2)} \frac{K}{\rho} \right], \end{aligned}$$

and $\frac{\partial V^{\mathcal{SP}}(q)}{\partial q_I^A} = 0$ gives $q_I^A = \frac{\beta_1(\mu_2)}{\beta_1(\mu_2)-1} \left(\frac{C}{K} \right)$.

The second order partial derivatives of $V^{\mathcal{SP}}(q)$ with respect to q_I^I is:

$$
\begin{aligned}
\frac{\partial^2 V^{\mathcal{SP}}(q)}{\partial {q_I^I}^2} =& q^{\beta_1(\mu_1)}(\beta_1(\mu_2)-\beta_1(\mu_1)-1)(\beta_1(\mu_2)-\beta_1(\mu_1))(q_I^I)^{\beta_1(\mu_2)-\beta_1(\mu_1)-2} \\
& \times (q_I^A)^{-\beta_1(\mu_2)}\left(\frac{Kq_I^A-C}{\rho}\right) - q^{\beta_1(\mu_1)}(\beta_1(\mu_1)+1)\beta_1(\mu_1)(q_I^I)^{-\beta_1(\mu_1)-2}I \\
=& (q_I^I)^{-\beta_1(\mu_1)-2}\Bigg[q^{\beta_1(\mu_1)}(\beta_1(\mu_2)-\beta_1(\mu_1)-1)(\frac{q_I^I}{q_I^A})^{\beta_1(\mu_2)}(\beta_1(\mu_2)-\beta_1(\mu_1)) \\
& \times \left(\frac{Kq_I^A-C}{\rho}\right) - q^{\beta_1(\mu_1)}(\beta_1(\mu_1)+1)\beta_1(\mu_1)I\Bigg] \\
=& (q_I^I)^{-\beta_1(\mu_1)-2}\Bigg[q^{\beta_1(\mu_1)}(\beta_1(\mu_2)-\beta_1(\mu_1)-1)(\beta_1(\mu_2)-\beta_1(\mu_1)) \\
& \times \frac{\beta_1(\mu_1)I}{\beta_1(\mu_1)-\beta_1(\mu_2)} - q^{\beta_1(\mu_1)}(\beta_1(\mu_1)+1)\beta_1(\mu_1)I\Bigg] \\
=& -(q_I^I)^{-\beta_1(\mu_1)-2}\left[q^{\beta_1(\mu_1)}\beta_1(\mu_1)\beta_1(\mu_2)I\right] < 0.
\end{aligned}
$$

The second order partial derivatives of $V^{\mathcal{SP}}(q)$ with respect to q_I^A is:

$$
\begin{aligned}
\frac{\partial^2 V^{\mathcal{SP}}(q)}{\partial {q_I^A}^2} =& q^{\beta_1(\mu_1)}(q_I^I)^{\beta_1(\mu_2)-\beta_1(\mu_1)}\frac{K}{\rho}(1-\beta_1(\mu_2))(-\beta_1(\mu_2))(q_I^A)^{-\beta_1(\mu_2)-1} \\
& -(\beta_1(\mu_2)+1)\beta_1(\mu_2)q^{\beta_1(\mu_2)}(q_I^I)^{\beta_1(\mu_2)-\beta_1(\mu_2)}(q_I^A)^{-\beta_1(\mu_2)-2}\left(\frac{C}{\rho}\right) \\
=& q^{\beta_1(\mu_1)}(q_I^I)^{\beta_1(\mu_2)-\beta_1(\mu_1)}(q_I^A)^{-\beta_1(\mu_2)-2}\Bigg[\frac{K}{\rho}(\beta_1(\mu_2)-1)(\beta_1(\mu_2))q_I^A \\
& -(\beta_1(\mu_2)+1)\beta_1(\mu_2)\left(\frac{C}{\rho}\right)\Bigg] \\
=& -q^{\beta_1(\mu_1)}(q_I^I)^{\beta_1(\mu_2)-\beta_1(\mu_1)}(q_I^A)^{-\beta_1(\mu_2)-2}\beta_1(\mu_2)\left(\frac{C}{\rho}\right) < 0.
\end{aligned}
$$

The derivatives of $V^{\mathcal{SP}}(q)$ with respect to q_I^A first, then w.r.t q_I^I is

$$\begin{aligned}\frac{\partial^2 V^{\mathcal{SP}}(q)}{\partial q_I^I \partial q_I^A} =&(\beta_1(\mu_2)-\beta_1(\mu_1))(q_I^I)^{\beta_1(\mu_2)-\beta_1(\mu_1)-1}(q_I^A)^{-\beta_1(\mu_2)}\\ &\times\left[q^{\beta_1(\mu_1)}\frac{K}{\rho}(1-\beta_1(\mu_2))+\beta_1(\mu_2)q^{\beta_1(\mu_1)}\frac{1}{q_I^A}\left(\frac{C}{\rho}\right)\right]\\ =&(\beta_1(\mu_2)-\beta_1(\mu_1))(q_I^I)^{\beta_1(\mu_2)-\beta_1(\mu_1)-1}(q_I^A)^{-\beta_1(\mu_2)}\\ &\times\left[q^{\beta_1(\mu_1)}\frac{K}{\rho}(1-\beta_1(\mu_2))+\beta_1(\mu_2)q^{\beta_1(\mu_1)}\frac{\beta_1(\mu_2)-1}{\beta_1(\mu_2)}\frac{K}{\rho}\right]\\ =&0.\end{aligned}$$

Thus

$$D=\frac{\partial^2 V^{\mathcal{SP}}(q)}{\partial {q_I^I}^2}\frac{\partial^2 V^{\mathcal{SP}}(q)}{\partial {q_I^A}^2}-\left(\frac{\partial^2 V^{\mathcal{SP}}(q)}{\partial q_I^I \partial q_I^A}\right)^2>0.$$

Hence it is proved that the value of project ran by the social planner $V^{\mathcal{SP}}(q)$ is maximized if the R&D process starts at q_I^I and the quality standard is set to be q_I^A.

E PROOF OF PROPOSITION 6

Rewrite q_I^I by substituting q_I^A,

$$\begin{aligned}q_I^I &= q_I^A\left(\frac{\beta_1(\mu_1)I}{\beta_1(\mu_1)-\beta_1(\mu_2)}\frac{\rho}{Kq_I^A-C}\right)^{1/\beta_1(\mu_2)}\\ &= q_I^A\left(\frac{\beta_1(\mu_1)I}{\beta_1(\mu_1)-\beta_1(\mu_2)}\frac{\rho(\beta_1-1)}{C}\right)^{1/\beta_1(\mu_2)}\\ &=\frac{\beta_1(\mu_2)}{\beta_1(\mu_2)-1}\left(\frac{C}{K}\right)\left(\frac{\beta_1(\mu_1)I}{\beta_1(\mu_1)-\beta_1(\mu_2)}\frac{\rho(\beta_1-1)}{C}\right)^{1/\beta_1(\mu_2)}.\end{aligned}$$

Then rewrite q_c^I by substituting q_c^A,

$$\begin{aligned}q_c^I &= q_c^A\left(\frac{\beta_1(\mu_1)}{\beta_1(\mu_1)-\beta_1(\mu_2)}\frac{\rho I}{R-C}\right)^{1/\beta_1(\mu_2)}\\ &=\frac{\beta_1(\mu_1)}{\beta_1(\mu_1)-1}\left(\frac{R}{K}\right)\left(\frac{\beta_1(\mu_1)}{\beta_1(\mu_1)-\beta_1(\mu_2)}\frac{\rho I}{R-C}\right)^{1/\beta_1(\mu_2)}.\end{aligned}$$

Divide q_c^I by q_I^I,

$$W(C)=\frac{q_c^I}{q_I^I}=\frac{\beta_1(\mu_1)(\beta_1(\mu_2)-1)}{\beta_1(\mu_2)(\beta_1(\mu_1)-1)}\left(\frac{R}{C}\right)\left(\frac{C}{R-C}\frac{1}{\beta_1(\mu_2)-1}\right)^{1/\beta_1(\mu_2)}.$$

Take the first order derivative of $W(C)$ with respect to C,

$$\frac{\partial W(C)/\partial C}{A} = \left(-\frac{1}{C^2}\right)\left(\frac{C}{R-C}\right)^{1/\beta_1(\mu_2)} + \frac{1}{C}\frac{1}{\beta_1(\mu_2)}\left(\frac{C}{R-C}\right)^{1/\beta_1(\mu_2)-1}\frac{R}{(R-C)^2},$$

where

$$A = \frac{\beta_1(\mu_1)(\beta_1(\mu_2)-1)}{\beta_1(\mu_2)(\beta_1(\mu_1)-1)}\left(\frac{1}{\beta_1(\mu_2)-1}\right)^{1/\beta_1(\mu_2)}.$$

And $\frac{\partial W(C)/\partial C}{A} = 0$ gives

$$C = \left(\frac{\beta_1(\mu_2)-1}{\beta_1(\mu_2)}\right)R.$$

The ratio $\frac{q_c^I}{q_I^I}$ can be treated as a function of C. Consider the two functions $f_1(C) = \frac{1}{C}$ and $f_2(C) = \left(\frac{C}{R-C}\right)^{1/\beta_1(\mu_2)}$. $f_1(C)$ is convex for every value of C. For computational convenience, denote $\alpha = 1/\beta_1(\mu_2)$ and

$$\begin{aligned}\frac{\partial^2 f_2(C)}{\partial C^2} &= \frac{\partial^2 \left(\frac{C}{R-C}\right)^\alpha}{\partial C^2}\\ &= R\alpha\left\{C^{\alpha-2}(R-C)^{-\alpha-2}\left[(\alpha-1)(R-C)+C((\alpha+1)\right]\right\}\\ &= R\alpha\left\{C^{\alpha-2}(R-C)^{-\alpha-2}\left[2C-(1-\alpha)R\right]\right\}.\end{aligned}$$

When $C = \left(\frac{\beta_1(\mu_2)-1}{\beta_1(\mu_2)}\right)R$,

$$\begin{aligned}&\frac{\partial^2 f_2(C)}{\partial C^2}\\ &= R\alpha\left\{C^{\alpha-2}(R-C)^{-\alpha-2}(1-\alpha)R\right\}\\ &= R\alpha\left\{C^{\alpha-2}(R-C)^{-\alpha-2}\left(\frac{\beta_1(\mu_2)-1}{\beta_1(\mu_2)}\right)R\right\} > 0.\end{aligned}$$

Hence, the function $f_2(C)$ is convex on the point $C = \left(\frac{\beta_1(\mu_2)-1}{\beta_1(\mu_2)}\right)R$.

Since both functions $f_1(C)$ and $f_2(C)$ are non-negative and convex, the product of two non-negative and convex functions is also a convex function. See Appendix.

Because $W\left[\left(\frac{\beta_1(\mu_2)-1}{\beta_1(\mu_2)}\right)R\right] = \frac{\beta_1(\mu_1)}{\beta_1(\mu_1)-1} > 1$, the ratio $W(C) = \frac{q_c^I}{q_I^I}$ is larger than 1 when it takes the minimum value, which shows that $q_c^I > q_I^I$.

F PROOF OF PROPOSITION 7

If $q_c^A < q_I^A$, (see Proposition 3 and eq. (25), the following condition must be satisfied

$$R < \frac{(\beta_1(\mu_1) - 1)\beta_1(\mu_2)}{(\beta_1(\mu_2) - 1)\beta_1(\mu_1)} C.$$

However, inequality (29) will also be satisfied at the same time. Thus,

$$\frac{\beta_1(\mu_1)}{\beta_1(\mu_1) - \beta_1(\mu_2)} \rho I + C < R < \frac{(\beta_1(\mu_1) - 1)\beta_1(\mu_2)}{(\beta_1(\mu_2) - 1)\beta_1(\mu_1)} C. \tag{F.1}$$

Now, inequality (F.1) only holds if the sunk cost of the project I satisfies,

$$\frac{\beta_1(\mu_1)}{\beta_1(\mu_1) - \beta_1(\mu_2)} \rho I + C < \frac{(\beta_1(\mu_1) - 1)\beta_1(\mu_2)}{(\beta_1(\mu_2) - 1)\beta_1(\mu_1)} C,$$

or

$$I < \frac{(\beta_1(\mu_1) - \beta_1(\mu_2))^2 C}{\beta_1(\mu_1)^2 (\beta_1(\mu_2) - 1)\rho}.$$

Since it is by assumption that $q_c^I < q_c^A$ and it has been proved that $q_I^I < q_c^I$, $q_I^I < q_c^I < q_c^A < q_I^A$.

G PROOF OF PROPOSITION 8

If $q_I^A \leq q_c^A$, (see Proposition 3 and eq. (25)), the following condition must be satisfied

$$R \geq \frac{(\beta_1(\mu_1) - 1)\beta_1(\mu_2)}{(\beta_1(\mu_2) - 1)\beta_1(\mu_1)} C.$$

Since by assumption, $q_c^I \leq q_c^A$, the following condition holds

$$R > \frac{\beta_1(\mu_1)}{\beta_1(\mu_1) - \beta_1(\mu_2)} \rho I + C.$$

If

$$\frac{\beta_1(\mu_1)}{\beta_1(\mu_1) - \beta_1(\mu_2)} \rho I + C < \frac{(\beta_1(\mu_1) - 1)\beta_1(\mu_2)}{(\beta_1(\mu_2) - 1)\beta_1(\mu_1)} C, \tag{G.1}$$

the sunk cost I will be satisfying

$$I < \frac{(\beta_1(\mu_1) - \beta_1(\mu_2))^2 C}{\beta_1(\mu_1)^2 (\beta_1(\mu_2) - 1)\rho}.$$

Hence

$$R \geq \frac{(\beta_1(\mu_1)-1)\beta_1(\mu_2)}{(\beta_1(\mu_2)-1)\beta_1(\mu_1)}C.$$

Next we prove that there is a unique R_c which makes $q_c^I = q_I^A$. Take the first order derivative of q_c^I with respect to R, we will find that $\frac{\partial q_c^I}{\partial R} < 0$ if $R \in \left[\frac{(\beta_1(\mu_1)-1)\beta_1(\mu_2)}{(\beta_1(\mu_2)-1)\beta_1(\mu_1)}C, \frac{\beta_1(\mu_2)}{\beta_1(\mu_2)-1}C\right)$ and $\frac{\partial q_c^I}{\partial R} \geq 0$ if $R \in \left[\frac{\beta_1(\mu_2)}{\beta_1(\mu_2)-1}C, \infty\right)$.

Moreover,

$$\frac{\beta_1(\mu_2)}{\beta_1(\mu_2)-1}C - \frac{(\beta_1(\mu_1)-1)\beta_1(\mu_2)}{(\beta_1(\mu_2)-1)\beta_1(\mu_1)}C = \frac{\beta_1(\mu_2)C}{(\beta_1(\mu_2)-1)\beta_1(\mu_1)} > 0.$$

When $R = \frac{(\beta_1(\mu_1)-1)\beta_1(\mu_2)}{(\beta_1(\mu_2)-1)\beta_1(\mu_1)}C$,

$$\begin{aligned} q_c^I &= \frac{\beta_1(\mu_2)}{\beta_1(\mu_2)-1}\frac{C}{K}\left[\frac{\beta_1(\mu_1)}{\beta_1(\mu_1)-\beta_1(\mu_2)}\frac{\rho I}{R-C}\right]^{1/\beta_1(\mu_2)} \\ &= q_I^A\left[\frac{\beta_1(\mu_1)}{\beta_1(\mu_1)-\beta_1(\mu_2)}\frac{\rho I}{R-C}\right]^{1/\beta_1(\mu_2)}. \end{aligned}$$

By assumption, $\left[\frac{\beta_1(\mu_1)}{\beta_1(\mu_1)-\beta_1(\mu_2)}\frac{\rho I}{R-C}\right]^{1/\beta_1(\mu_2)} < 1$, thus $q_c^I < q_I^A$.

Since

$$q_c^I = \frac{\beta_1(\mu_1)}{\beta_1(\mu_1)-1}\frac{R}{K}\left[\frac{\beta_1(\mu_1)}{\beta_1(\mu_1)-\beta_1(\mu_2)}\frac{\rho I}{R-C}\right]^{1/\beta_1(\mu_2)},$$

to see what happen to q_c^I when R goes to infinity, we compute $\lim_{R\to+\infty}\frac{R}{(R-C)^{1/\beta_1(\mu_2)}}$ by using L'Hospital's Rule and $\lim_{R\to\infty}\frac{R}{(R-C)^{1/\beta_1(\mu_2)}} = \lim_{R\to\infty}\beta_1(\mu_2)(R-C)^{1-1/\beta_1(\mu_2)} = +\infty > q_I^A$. Thus there is a unique R_c makes $q_c^I = q_I^A$.

It is by assumption that $q_c^I < q_I^A$ and $q_I^I < q_c^I$. In conclusion, $q_I^I < q_c^I < q_I^A \leq q_c^A$ on $\left[\frac{(\beta_1(\mu_1)-1)\beta_1(\mu_2)}{(\beta_1(\mu_2)-1)\beta_1(\mu_1)}C, R_c\right)$ and $q_I^I < q_I^A \leq q_c^I < q_c^A$ on $\left[R_c, \infty\right)$.

If inequality (G.1) is changed to be

$$\frac{\beta_1(\mu_1)}{\beta_1(\mu_1)-\beta_1(\mu_2)}\rho I + C \geq \frac{(\beta_1(\mu_1)-1)\beta_1(\mu_2)}{(\beta_1(\mu_2)-1)\beta_1(\mu_1)}C,$$

the sunk cost I will be satisfying

$$I \geq \frac{(\beta_1(\mu_1)-\beta_1(\mu_2))^2 C}{\beta_1(\mu_1)^2(\beta_1(\mu_2)-1)\rho}.$$

Hence

$$R \geq \frac{\beta_1(\mu_1)}{\beta_1(\mu_1) - \beta_1(\mu_2)} \rho I + C.$$

There are only two differences in the conclusion which is to change the start value to $R = \frac{\beta_1(\mu_1)}{\beta_1(\mu_1)-\beta_1(\mu_2)}\rho I + C$ and denote the critical value that makes $q_c^I = q_I^A$ as R_{cc}. The rest of the proofs are the same. The final conclusion will be $q_I^I < q_c^I < q_I^A \leq q_c^A$ on $\left[\frac{\beta_1(\mu_1)}{\beta_1(\mu_1)-\beta_1(\mu_2)}\rho I + C, R_{cc}\right)$ and $q_I^I < q_I^A \leq q_c^I < q_c^A$ on $\left[R_{cc}, \infty\right)$.

H PROOF OF PROPOSITION 10

Dependent on the initial value of product's quality q, the total value of the projects are different. When $q < q_I^I < q_c^I$, the total value of both projects are

$$V^{\mathcal{PF}}(q) + V^{\mathcal{HA}}(q) = \left(\frac{q}{q_c^I}\right)^{\beta_1(\mu_1)} \left[\left(\frac{q_c^I}{q_c^A}\right)^{\beta_1(\mu_2)} \left(\frac{Kq_c^A - C}{\rho}\right) - I\right]$$

and

$$V^{\mathcal{SP}}(q) = \left(\frac{q}{q_I^I}\right)^{\beta_1(\mu_1)} \left[\left(\frac{q_I^I}{q_I^A}\right)^{\beta_1(\mu_2)} \left(\frac{Kq_I^A - C}{\rho}\right) - I\right].$$

q_I^I and q_c^I are the optimal investment thresholds of both projects and they solve the maximization problems of the social planner's project and the pharmaceutical company's project respectively. However, the maximization problem of the social planner's project is an unconstrained problem while the maximization problem of the pharmaceutical company's problem is limited by the quality standard set by the health authority. In this case, the option value of the pharmaceutical company is not fully captured which leads to a lower project value. Thus the total value of the decentralized project is lower than that of the social planner's project.

When $q_I^I < q_c^I < q$, both projects start immediately. The total value of both projects are

$$V^{\mathcal{PF}}(q) + V^{\mathcal{HA}}(q) = \left(\frac{q}{q_c^A}\right)^{\beta_1(\mu_2)} \left(\frac{Kq_c^A - C}{\rho}\right) - I,$$

and

$$V^{\mathcal{SP}}(q) = \left(\frac{q}{q_I^A}\right)^{\beta_1(\mu_2)} \left(\frac{Kq_I^A - C}{\rho}\right) - I,$$

respectively.

In general, the total value of each project can be represented by the following function

$$NPV(x) = \left(\frac{q}{x}\right)^{\beta_1(\mu_2)} \left(\frac{Kx - C}{\rho}\right) - I,$$

where $x > 0$.

Take the first order derivative of $NPV(x)$ w.r.t. x,

$$NPV^{'}(x) = q^{\beta_1(\mu_2)} x^{-\beta_1(\mu_2)-1} \left((1-\beta_1(\mu_2))\frac{Kx}{\rho} + \frac{C}{\rho}\beta_1(\mu_2)\right).$$

It is shown that $NPV^{'}(x) > 0$ if $0 < x < \frac{\beta_1(\mu_2)}{\beta_1(\mu_2)-1}\frac{C}{K} = q_I^A$, $NPV^{'}(x) = 0$ if $x = q_I^A$ and $NPV^{'}(x) < 0$ if $x > q_I^A$.

Now take the second order derivative of $NPV(x)$ w.r.t. x,

$$NPV^{''}(x) = q^{\beta_1(\mu_2)}[-\beta_1(\mu_2)]x^{-\beta_1(\mu_2)-2} \left[(1-\beta_1(\mu_2))x\frac{K}{\rho} + \frac{C}{\rho}(\beta_1(\mu_2)+1)\right].$$

It is shown that $NPV^{''}(x) > 0$ if $x > \frac{\beta_1(\mu_2)+1}{\beta_1(\mu_2)-1}\frac{C}{K} = q_I^A + \frac{C}{(\beta_1(\mu_2)-1)K}$, $NPV^{'}(x) = 0$ if $x = \frac{\beta_1(\mu_2)+1}{\beta_1(\mu_2)-1}\frac{C}{K}$ and $NPV^{'}(x) < 0$ if $0 < x < \frac{\beta_1(\mu_2)+1}{\beta_1(\mu_2)-1}\frac{C}{K}$.

In conclusion, $NPV(x)$ is an increasing concave function on $(0, q_I^A)$ and reaches its maximum at q_I^A. Then it decreases and remains being concave on $(q_I^A, q_I^A + \frac{C}{(\beta_1(\mu_2)-1)K})$ then it keeps decreasing and being convex on $[q_I^A + \frac{C}{(\beta_1(\mu_2)-1)K}, \infty)$.

Thus,

$$\left(\frac{q}{q_I^A}\right)^{\beta_1(\mu_2)} \left(\frac{Kq_I^A - C}{\rho}\right) - I > \left(\frac{q}{q_c^A}\right)^{\beta_1(\mu_2)} \left(\frac{Kq_c^A - C}{\rho}\right) - I.$$

And it is proved that the value of the social planner's project is strictly larger than that of the decentralized project when $q_I^I < q_c^I < q$.

When $q_I^I < q < q_c^I$, the social planner's project starts immediately while the decentralized project has not started yet. The total value of both projects are

$$V^{\mathcal{PF}}(q) + V^{\mathcal{HA}}(q) = \left(\frac{q}{q_c^I}\right)^{\beta_1(\mu_1)} \left[\left(\frac{q_c^I}{q_c^A}\right)^{\beta_1(\mu_2)} \left(\frac{Kq_c^A - C}{\rho}\right) - I\right], \tag{H.1}$$

and

$$V^{\mathcal{SP}}(q) = \left(\frac{q}{q_I^A}\right)^{\beta_1(\mu_2)} \left(\frac{Kq_I^A - C}{\rho}\right) - I. \tag{H.2}$$

Since the total value of the social planner's project is larger than that of the decentralized project when $q \in [0, q_I^I) \cup [q_c^I, +\infty]$, if $V^{\mathcal{SP}}(q)$ is more convex on $[q_I^I, q_c^I)$, $V^{\mathcal{SP}}(q) > V^{\mathcal{PF}}(q) + V^{\mathcal{HA}}(q)$.

Take the second order partial derivatives of eq. (H.1) with respect to q and

$$
\begin{aligned}
\left[V^{\mathcal{PF}}(q) + V^{\mathcal{HA}}(q)\right]'' &= \left(\frac{q}{q_c^I}\right)^{\beta_1(\mu_1)} \left[\left(\frac{q_c^I}{q_c^A}\right)^{\beta_1(\mu_2)} \left(\frac{Kq_c^A - C}{\rho}\right) - I\right] \\
&\quad \times \beta_1(\mu_2)(\beta_1(\mu_2) - 1)\frac{1}{q^2} \\
&< \left(\frac{q}{q_c^I}\right)^{\beta_1(\mu_2)} \left[\left(\frac{q_c^I}{q_c^A}\right)^{\beta_1(\mu_2)} \left(\frac{Kq_c^A - C}{\rho}\right) - I\right] \\
&\quad \times \beta_1(\mu_2)(\beta_1(\mu_2) - 1)\frac{1}{q^2} \\
&< \left(\frac{q}{q_c^A}\right)^{\beta_1(\mu_2)} \left(\frac{Kq_c^A - C}{\rho}\right) \beta_1(\mu_2)(\beta_1(\mu_2) - 1)\frac{1}{q^2} \\
&< \left(\frac{q}{q_I^A}\right)^{\beta_1(\mu_2)} \left(\frac{Kq_I^A - C}{\rho}\right) \beta_1(\mu_2)(\beta_1(\mu_2) - 1)\frac{1}{q^2} \\
&= \left[V^{\mathcal{SP}}(q)\right]''.
\end{aligned}
$$

Hence we have proved that $V^{\mathcal{SP}}(q)$ is more convex on $[q_I^I, q_c^I)$. Thus the project value of the social planner's project is larger than that of the decentralized one.
